# Laser spectroscopy for breath analysis: towards clinical implementation

**DOI:** 10.1007/s00340-018-7030-x

**Published:** 2018-07-28

**Authors:** Ben Henderson, Amir Khodabakhsh, Markus Metsälä, Irène Ventrillard, Florian M. Schmidt, Daniele Romanini, Grant A. D. Ritchie, Sacco te Lintel Hekkert, Raphaël Briot, Terence Risby, Nandor Marczin, Frans J. M. Harren, Simona M. Cristescu

**Affiliations:** 1 Trace Gas Research Group, Molecular and Laser Physics, IMM, Radboud University, 6525 AJ Nijmegen, The Netherlands; 2 Department of Chemistry, University of Helsinki, PO Box 55, 00014 Helsinki, Finland; 3 University of Grenoble Alpes, CNRS, LIPhy, 38000 Grenoble, France; 4 Department of Applied Physics and Electronics, Umeå University, 90187 Umeå, Sweden; 5 University of Grenoble Alpes, CNRS, TIMC-IMAG, 38000 Grenoble, France; 6 Department of Chemistry, Physical and Theoretical Chemistry Laboratory, University of Oxford, South Parks Road, Oxford, OX1 3QZ UK; 7 Sensor Sense B.V, St. Agnetenweg 103, 6545 AV Nijmegen, The Netherlands; 8 Emergency Department and Mobile Intensive Care Unit, Grenoble University Hospital, Grenoble, France; 9 Department of Environmental Health and Engineering, Bloomberg School of Public Health, The Johns Hopkins University, Baltimore, USA; 10 Section of Anaesthetics, Pain Medicine and Intensive Care, Department of Surgery and Cancer, Faculty of Medicine, Imperial College London, London, UK; 11 Centre of Anaesthesia and Intensive Care, Semmelweis University, Budapest, Hungary

## Abstract

Detection and analysis of volatile compounds in exhaled breath represents an attractive tool for monitoring the metabolic status of a patient and disease diagnosis, since it is non-invasive and fast. Numerous studies have already demonstrated the benefit of breath analysis in clinical settings/applications and encouraged multidisciplinary research to reveal new insights regarding the origins, pathways, and pathophysiological roles of breath components. Many breath analysis methods are currently available to help explore these directions, ranging from mass spectrometry to laser-based spectroscopy and sensor arrays. This review presents an update of the current status of optical methods, using near and mid-infrared sources, for clinical breath gas analysis over the last decade and describes recent technological developments and their applications. The review includes: tunable diode laser absorption spectroscopy, cavity ring-down spectroscopy, integrated cavity output spectroscopy, cavity-enhanced absorption spectroscopy, photoacoustic spectroscopy, quartz-enhanced photoacoustic spectroscopy, and optical frequency comb spectroscopy. A SWOT analysis (strengths, weaknesses, opportunities, and threats) is presented that describes the laser-based techniques within the clinical framework of breath research and their appealing features for clinical use.

## Introduction

Humans exhale hundreds of gases, including inorganic compounds (e.g., ammonia, nitric oxide, and hydrogen sulfide) and volatile organic compounds (VOCs), arising from normal body metabolism [1–4]. Human breath also contains exogenous compounds that originate from food and beverages or from current or previous environmental exposures [5]. Endogenous emissions reflect the changes in the internal metabolism when illness occur but can also vary in response to various (external) stimuli [6, 7]. Since the time of Hippocrates, some diseases have been recognized by their associated odor [8]. After Pauling’s breakthrough in 1971 [9], modern breath analysis has connected these odors with specific gas analytes (biomarkers) or a profile of VOCs. Although many exhaled compounds potentially reflect the physiological and pathophysiological conditions related to various diseases, only some VOCs have been established as biomarkers [10].

Several methods are currently employed for exhaled volatiles detection, among which mass spectrometry (e.g., gas chromatography mass spectrometry, GC-MS) is the most widely used analytical tool [11–14]. However, the GC-MS-based instruments are limited to laboratory settings, do not allow online sampling (i.e., exhaling directly into the instrument, usually using an inbuilt system or an external breath sampler for feedback exhaled airflow control, including a flowmeter) and have a relatively long analysis time (in the order of tens of minutes). For these reasons, extensive research has been conducted to develop alternate methods that can be used to perform rapid, sensitive, online analysis in a clinical setting, preferably for many volatile compounds simultaneously. Proton-transfer reaction mass spectrometry (PTR-MS) [15], selected ion flow-tube mass spectrometry (SIFT-MS) [16], gas chromatography combined with ion mobility spectrometry (GC-IMS) [17], and secondary electro-spray ionization mass spectrometry (SESI-MS) [18] are a few examples of online mass-spectrometric (MS) methods currently employed in breath analysis. In spite of these advances, there is a continuous need for miniaturized devices, in addition to the consideration of an affordable, accurate, and user-friendly instrument that should provide fast response, preferably in real time.

Compared to online mass spectrometry, laser-based spectroscopy can provide an accurate and precise quantitative analysis result, with an instrument that does not necessarily require an expert user. There are already a few optical breath analyzers commercially available for monitoring different biomarkers in clinical applications, e.g., non-dispersive infrared (NDIR) spectrometers for capnography and for measuring ^13^C in breath to diagnose *Helicobacter pylori* (the bacterium that generates gastric ulcers and gastric cancers) [19], an infrared spectrometer for monitoring of CH_4_ in breath accompanied by electrochemical sensors to detect H_2_ and O_2_ for diagnosis of gastrointestinal disorders [20], and a cavity ring-down spectroscopy system for detection of breath acetone which correlates with abnormal metabolic status, such as diabetes [21]. These successful implementations of optical breath analyzers in real-life clinical applications show the bright future of these systems, especially those using lasers as light sources.

Laser spectroscopy can provide real-time (sub-second time resolution and breath-cycle resolved) detection of a single or several volatile compounds, with detection limits ranging from the part-per-million (ppm, µmol /mol) to less than part-per-billion (ppb, nmol /mol). Fast online sampling, combined with real-time detection, eliminates the need for collection and storage, which constituted a potential source of errors because of the risk for contamination and dilution of the breath sample. Furthermore, breath-cycle resolved sampling adds significant advantages such as, resolving the different respiratory phases and continuous measurements of the volatiles/biomarkers present in these different phases, and the possibility to couple and validate physiological and gas exchange models. The high precision, absolute accuracy, and breath-cycle resolved sampling allow much more data to be collected and very detailed biomarker studies to be performed. Hence, small differences can be resolved and gas exchange processes in the respiratory tract can be characterized, allowing us to gain a better understanding of the origin and chemical pathways of the (potential) biomarkers. This, in turn, provides a clearer picture about which biomarkers really correlate with diseases, which has been (and still is) one of the main difficulties researchers face in the field of breath research. On the other hand, the future of breath analysis could lie in monitoring changes in biomarker concentrations compared to control or baseline values, especially to determine the specific response to interventions, such as a pathogen attack, drug treatment, or the intake of isotope-labeled substances.

In general, a detection limit of 100 ppt can be considered sufficient for almost any breath analysis application. The ambient levels of most VOCs will be higher than this concentration, and analyzing lower concentrations from exhaled breath has questionable utility. There have been several approaches proposed to correct for the ambient background contribution in breath measurements [22], but none of these are entirely reliable. If the concentration of the target species in breath is high (as, for e.g., acetone, isoprene, ammonia, methane, and carbon monoxide), a more modest detection limit is obviously adequate. For real-time measurements, an acquisition rate between 1 and 10 Hz should be satisfactory in all cases. This will allow a complete characterization of individual breath cycles and resolve any disparity between the analyte behavior and a standard capnogram (representation of the partial pressure of CO_2_ versus time).

Several reviews covering laser spectroscopic techniques for breath gas analysis are available in the literature [22–26]. Together, these reviews provide a comprehensive overview of near-infrared (near-IR) and mid-IR laser-based breath analyzers using various spectroscopic methods, their challenges, and perspectives. Lourenco and Turner compare laser-based methods with other detection techniques employed in the field, discuss issues concerning breath sampling and biochemistry, and highlight the potential of breath analysis for disease diagnosis [22]. The status of optical spectroscopy based on mid-IR quantum and interband cascade lasers for detection of different biomarkers in breath is reviewed by Risby and Tittel [23]. The emphasis in the review by Stacewicz et al. [24] lies on detection of biomarkers using three different absorption spectroscopy techniques. Detailed technical descriptions of the experimental setups, which were developed by the authors, are provide in [26]. Wang and Sahay [25] explain the basic working principles of different laser spectroscopic techniques, and present promising biomarker molecules that can be targeted by laser spectroscopy, as well as their diagnostic significance. Next to the technological developments, several other considerations need to be taken into account for clinical applications of breath analysis: issues concerning sample collection and measurement, interferences, ambient air correction, etc. All these aspects have been discussed in details in the review articles [22, 23, 27]; however, few considerations regarding these are discussed here as well.

This work presents an update of the current status of the optical methods for breath analysis over the last decade and reviews the clinical applications of promising laser spectroscopic methods: tunable diode laser absorption spectroscopy (TDLAS), cavity ring-down spectroscopy (CRDS), integrated cavity output spectroscopy (ICOS)/cavity-enhanced absorption spectroscopy (CEAS), photoacoustic spectroscopy (PAS), quartz-enhanced photoacoustic spectroscopy (QEPAS), and optical frequency comb (OFC) spectroscopy. The selection of these techniques was based on their maturity and high potential for clinical applications, as well as the frequency in which they have been applied in breath research. Some of the most recent applications (from the past 5 years) of these techniques have been included to show the progress made from the laboratory demonstrator to commercial instruments used in clinical settings. Furthermore, the present review refers exclusively to the use of near and mid-IR sources in combination with the above techniques. A SWOT analysis (strengths, weaknesses, opportunities, and threats) is provided for the laser-based methods within the clinical framework of breath research.

Our hope is that the laser-based spectroscopy community recognizes the potential of these methods in breath research. Although, no technique is perfect, optical spectroscopy has appealing features for clinical use that are presented in this article. By recognizing and utilizing its specific strengths, laser spectroscopy could provide a strong contribution to this rapidly evolving field.

## Optical spectroscopy—basic principles and overview

The most common optical method used for exhaled breath analysis is ro-vibrational absorption spectroscopy, primarily because it affords selective determination of absolute concentrations. Normally, the analysis is based on the measurement of either fundamental (mid-IR, 3–10 µm) or first overtone (near-IR, 1–2 µm) vibrational transitions. In certain exceptional cases, electronic transitions in the visible or ultraviolet range can also be probed [25]. Emission transitions can also be exploited in some rare instances. A notable example is the very successful test for the diagnosis and monitoring of airway inflammation, based on detection of exhaled NO [28]. Measurement based on chemiluminescence is the gold standard for the F_E_NO (fractional exhaled NO) analysis. The reaction of NO with O_3_ produces an electronically excited state of nitrogen dioxide (NO_2_) which decays via emission at wavelengths above 600 nm [29].

The detection limit that is achieved in breath analysis by laser-based spectroscopy is determined by the sensitivity of the chosen technique. The sensitivity of an absorption measurement can be enhanced in two different ways: by either increasing the absorption path length or by increasing the signal-to-noise ratio. Only highly abundant compounds in breath can be quantified by a standard single-pass absorption measurement. Such an example is the ^13^CO_2_/^12^CO_2_ isotope ratio analysis with non-dispersive infrared (NDIR) spectroscopy. This technology is clinically exploited with success in the urea breath test for *H. pylori* diagnosis [30] and evaluating the activity of drug metabolizing enzymes, using stable isotope-labeled substrates [31]. To detect molecules present in breath at concentrations less than one part-per-million (ppm) levels, the absorption path length can be increased, by either using a multipass cell (MPC) or employing a resonant cavity-enhanced approach (e.g., CEAS, as defined above). In addition, the intensity of the optical beam can be modulated at a particular frequency to filter out instrumental noise with phase sensitive detection. Also, wavelength (WMS) and frequency modulation spectroscopic (FMS) methods are used for increasing the signal-to-noise ratio. Alternatively, photoacoustic spectroscopy can be employed; other than measuring the decrease of laser intensity by absorption, photoacoustic measures the result of absorption via an increase in acoustic intensity. Comparable sensitivities to absorption spectroscopy with short optical path length (tens of centimeters) can be achieved.

The concentrations of analytes that can be detected, using a single setup, depend normally on the type of laser source that is used. Current standard implementations of optical breath instrumentation use lasers with a limited tunability; this normally limits the number of analytes to a maximum of a few per instrument [32]. Wider tunable lasers can be used, such as optical frequency combs (OFCs). The spectral coverage will be increased significantly, as well as the number of simultaneously accessible analytes.

## Laser absorption spectroscopy

### Tunable diode laser and multipass absorption spectroscopy

Tunable diode laser absorption spectroscopy (TDLAS) is an umbrella term for the absorption techniques employing a single-mode semiconductor laser in a single- or multipass configuration and/or use modulation techniques for noise reduction. Thus, TDLAS comprises direct (single-pass) absorption spectroscopy, WMS, FMS, as well as path-length enhancement by various MPCs, such as a Herriott cells, White cells, or ring cells.

Typical light sources include distributed feedback (DFB) diode lasers (0.7–3 µm) and vertical-cavity surface-emitting lasers (VCSELs) in the visible and near-infrared spectral range, as well as DFB interband cascade lasers (ICLs) (3–6 µm), quantum cascade lasers (QCLs) (4–14 µm), and liquid nitrogen cooled lead salt tunable diode lasers in the mid-infrared [33]. These lasers allow mode-hop free wavelength tuning in a narrow wavelength range (1–5 cm^−1^) by varying the injection current or temperature, and usually enable simultaneous targeted detection of 1–3 species with low molecular weight. For extended multispecies detection, two (or several) narrowband lasers can be combined [34, 35]. Another approach is to use external-cavity diode lasers (ECDLs) and external-cavity quantum cascade lasers (EC-QCLs), where the laser is mounted in an optical cavity comprising a wavelength selective device, which provides a narrow linewidth and broadband wavelength tuning range (up to 100 cm^−1^) [36].

**Fig. 1:**
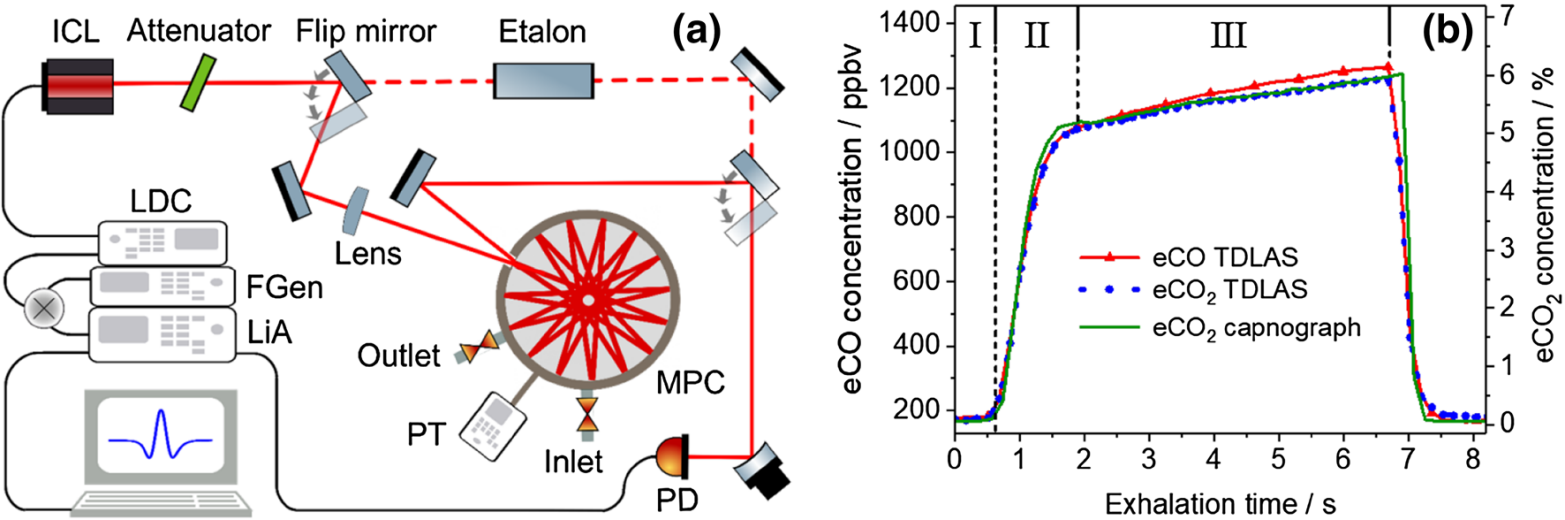
**a** Schematic drawing of a typical mid-infrared TDLAS setup employing an ICL, a circular, low-volume MPC, and WMS (with permission from [39]). LDC: laser diode controller, LiA: lock-in amplifier, FGen: function generator, PT: pressure transducer, PD: photodetector. **b** Real-time, exhalation profiles (breath cycles) of CO and CO_2_ measured with an EC-QCL, and a CO_2_ profile recorded with NDIR spectroscopy (capnography) (with permission from [36]). The three exhalation phases (dead space/airways, transition region, and alveolar region) are indicated

Setups based on direct absorption spectroscopy, which can only be used to detect the highly abundant species in breath carbon dioxide (CO_2_) and water vapor (H_2_O) [35–37], usually achieve a Noise Equivalent Absorption Sensitivity (NEAS) of 10^−3^–10^−5^ cm^−1^Hz^−1/2^. The online, real-time measurement and analysis of CO_2_ exhalation profiles (capnography) with NDIR spectroscopy is by far the most widely used application of breath analysis in clinical practice. Using modulation techniques and/or MPCs, the sensitivity can be increased by orders of magnitude. For example, sensitive detection of exhaled carbon monoxide (CO) was achieved with single-pass WMS [38]. The most sensitive and most frequently used TDLAS approach in breath gas analysis, however, is to combine WMS with multipass enhancement. With a typical NEAS of 10^−8^ cm^−1^Hz^−1/2^ and making use of the strong fundamental molecular transitions in the mid-infrared, detection limits in the low ppb range can be achieved for numerous important breath biomarkers (see Table 3). A typical experimental MPC/WMS setup is shown in Fig. 1a.

Real-time breath analysis is sometimes incompatible with the need for high sensitivities, which is usually achieved using long signal integration times (tens of seconds). In addition, the time resolution is also restricted by the large sample volume (around 0.5 L) of typical multipass cells, which limits the gas exchange time. Real-time breath gas analysis with sub-second acquisition times [34, 35, 38–40] is achieved for more abundant species, such as CO, e.g., using low-volume circular MPCs cells [36, 39]. An example of real-time detection of CO and CO_2_ exhalation profiles is shown in Fig. 1b.

Carbon monoxide, recently identified as a cellular signal molecule, is one of the biomarkers currently investigated [34, 36, 38, 39] with WMS and/or MPC enhanced TDLAS. Since the main endogenous CO sources are systemic and the metabolism of heme, this molecule is a potential biomarker for oxidative stress and respiratory diseases. As exposure to exogenous CO (smoking, air pollution) can obscure systemic contributions; the clinical significance of CO is currently under investigation. Compared to traditional electrochemical CO sensors (used to assess smoking status), TDLAS-based CO detection offers much better sensitivity, precision and time resolution, and thus is better suited to the study of CO physiology.

To summarize, TDLAS and multipass absorption spectroscopy offer good sensitivity and selectivity at high spectral and temporal resolution with potentially compact and affordable setups. Most of the implementations do not require calibration (using standard instrumentation with higher accuracy) to obtain quantitative results with high accuracy and precision. The method is suitable for the selective detection of exhaled breath biomarkers with end-tidal concentrations down to the low ppb range and rather strongly absorbing molecular transitions. For the main breath species and biomarkers with end-tidal levels around 1 ppm (CO, CH_4,_ NH_3_, and N_2_O), real-time detection and compact setups are within reach. Thus, in practice TDLAS is well-suited for targeted detection of one or a few such biomarkers, which so far has been one of the most successful application of breath tests in clinical applications.

### Absorption spectroscopy with external optical resonators

Many breath biomarkers are present at low ppb level or below and have weakly absorbing molecular transitions. Thus, to attain the necessary sensitivity and selectivity, ultra-sensitive cavity-enhanced absorption spectroscopy methods must be employed.

Compared to multipass cells, the absorption path length can be further increased by exploiting the principle of cavity-enhanced absorption (CEA). In CEA experiments, the breath sample is enclosed in a high-finesse optical cavity. In such systems, an optical cavity the absorption path length is dependent on the reflectivity of the cavity mirrors and can reach tens of kilometers in path length (with compact setups of ≈ 1 m long cavity), especially in wavelength regions where the mirror coating technology is advanced (i.e., the Telecom wavelength range). In the mid-infrared wavelength region, the mirror reflectivity is normally less impressive. However, this is compensated by the stronger absorption line strengths of the molecular transitions. In principle, all laser types used for TDLAS can also be used for CEA instruments.

CEA experiments can be found in many different settings. One of the most common approaches is to record the time evolution of the laser intensity inside the optical cavity and extract the time constant of decay. This approach is cavity ring-down spectroscopy (CRDS) and has been employed in a number of breath investigations [26]. Alternatively, the total amount of laser intensity that leaks out of the cavity can be recorded. Depending on the experimental details, the method is called either integrated cavity output spectroscopy (ICOS) or simply cavity-enhanced absorption spectroscopy (CEAS), depending on the author. The excitation of the optical cavity can be performed in various configurations and this leads to adapted acronyms: off-axis cavity-enhanced absorption spectroscopy (OA-CEAS) and optical feedback cavity-enhanced absorption spectroscopy (OF-CEAS). Typical sensitivities that can be achieved are in the 10^−10^–10^−11^ cm^−1^Hz^−1/2^ range.

#### Cavity-enhanced absorption spectroscopy

In many breath studies, “static” samples of breath are probed using instrumental averaging times ranging from several seconds to minutes. However, real-time laser spectroscopic measurements can yield important physiological and medical information of the main respiratory gases within a breath cycle.

A significant example is the recent work by Ciaffoni and co-workers [35] who have developed a sensor for the determination of oxygen consumption (VO_2_) on a breath-by-breath basis at the mouth of an intubated patient undergoing anesthesia and/or mechanical ventilation. The analyzer, termed a molecular flux sensor (MFS), uses diode laser absorption within an optical cavity [41] in combination with a state-of-the-art flow meter. The latter is instantly adaptable (10 ms resolution) to changing gas compositions. Here, a novel advance has been observed whereby the sensitivity of the CEAS measurement is improved by the application of broadband radiofrequency noise to the laser [42]. The MFS device has reduced the bias in flow sensing between inspiration and expiration from ~ 5% to < 0.2% and allows the quantification of clear and evident physiological responses to standard practice. This device allows the clinical studies necessary to understand the effects of a range of common interventions on VO_2_ in patients in various degrees of shock; these interventions include the use of fluids, blood transfusions, elevated oxygen, and vasoactive drugs. Such studies are a pre-requisite for the design of a goal-directed algorithm for an interventional clinical study using VO_2_ to titrate therapy.

**Fig. 2:**
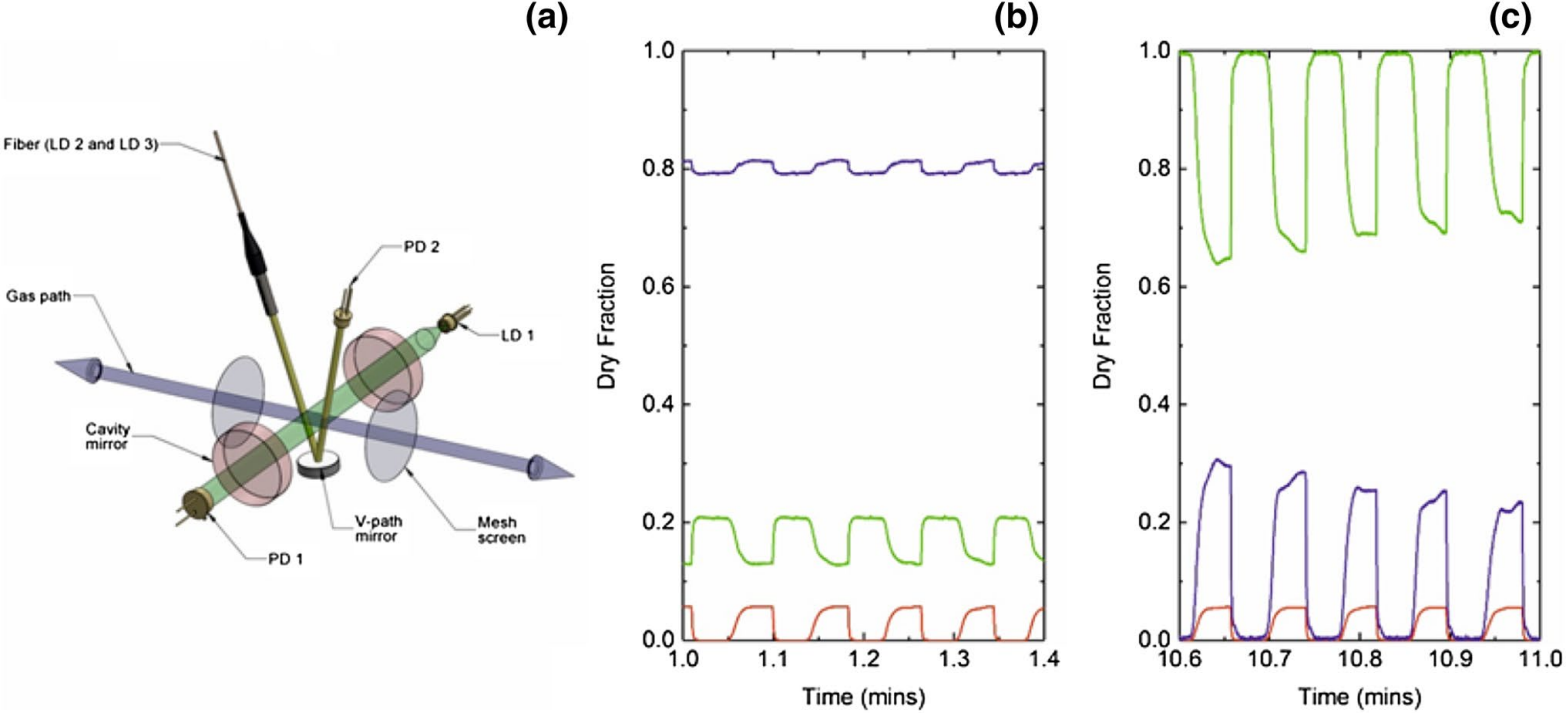
**a** Schematic of the multichannel absorption spectrometer and pneumotachograph within the MFS measurement head. The bi-directional gas path (blue) is shown along with the two mesh screens (gray), across which pressure drop is measured. Radiation used for probing O_2_ is injected into an optical cavity constructed from a pair of highly reflective mirrors (red) and collected by a photodiode (PD 1) positioned along the optical axis (green). Radiation probing CO_2_ and H_2_O vapor is launched into the v-path (yellow), reflected by a concave mirror, and collected by the photodiode (PD 2). The physical length of the optical cavity is commensurate with the diameter of a standard medical ventilation tube. **b, c** Dry gas fractions of N_2_ (purple), O_2_ (green), and CO_2_ (red) measured in real time (every 10 ms) as a participant breathes through the measurement head. The dry fraction of N_2_ is determined by the subtracting the dry fractions of O_2_ and CO_2_ from unity. **b** Data from an air breathing phase. **c** Data from the early stage of a nitrogen washout procedure

Most recently, the same group has recognized that the highly precise and time-resolved MFS data afford the opportunity to extract information pertaining to inhomogeneity in the lung from a period of steady breathing followed by a N_2_ multi-breath washout (MBW) phase (Fig. 2). The shape of the N_2_ MBW phase encodes a measure of the width of the alveolar ventilation to volume distribution, while the profiles, by which CO_2_ and O_2_ emerge during expiration, encode a measure of anatomic dead space and its distribution in the lung. Asymmetry between the CO_2_ and O_2_ “expirograms” reflects the distribution of ventilation to perfusion ratio in the lung, a consequence of differences in blood gas chemistry between the two [43].

Mountain et al. [44] have developed a model for lung inhomogeneity. The model has a parameter set that can be identified from measurements of respiratory gas flow, using the in-airway MFS. Key parameters that can be recovered are the standard deviations (*σ*) for the distributions: (1) in dead space (the volume of air which is inhaled that does not take part in the gas exchange, either because it remains in the conducting airways, or reaches alveoli that are not perfused or poorly perfused); (2) in fractional lung compliance (a measure of the lung’s ability to stretch and expand); and (3) in fractional vascular conductance (a measure of blood flow through the lung).

**Fig. 3:**
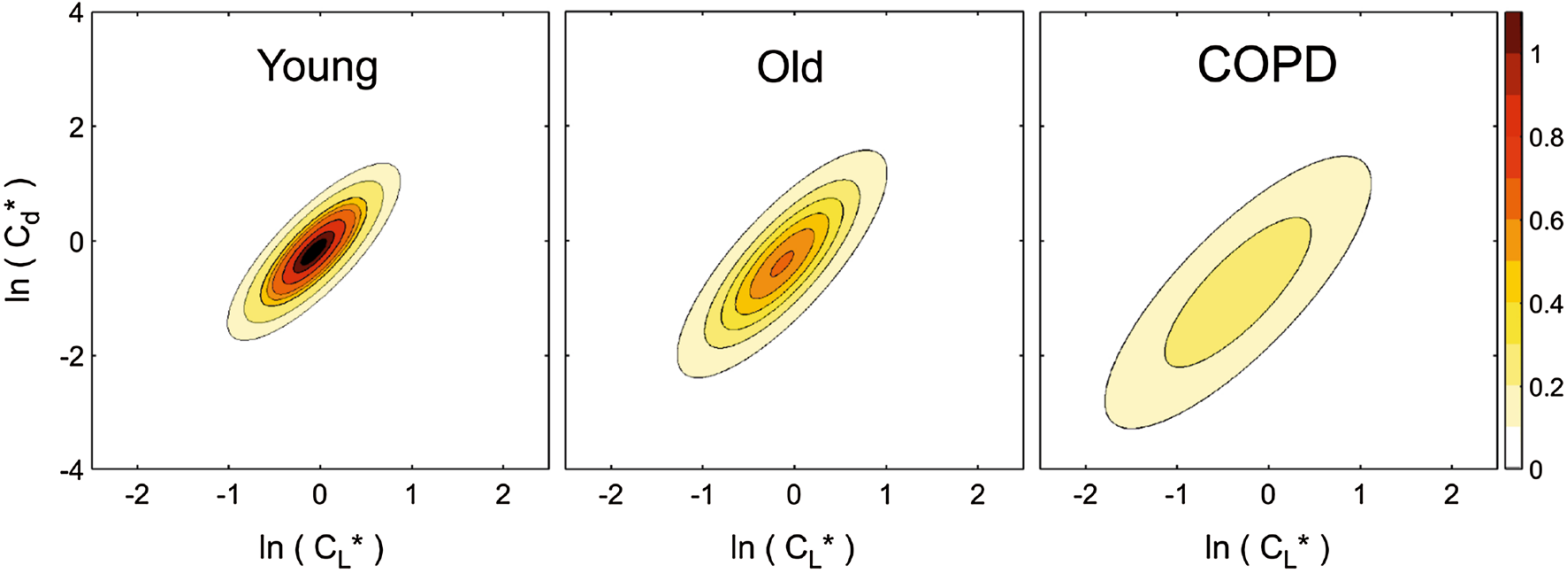
Contour plots for the bivariate log normal distributions for (standardized) compliance and conductance for three participant groups: young, old, and COPD. Contour intervals are values for the probability density function. The increased lung inhomogeneity for COPD cohort is readily apparent from the widths of the distribution. Further details can be found in Mountain et al. [44]

Notably, the MFS has been used to obtain pilot data from lung washouts, studying six healthy young participants (20–30 years), six healthy older participants (70–80 years), and six patients with chronic obstructive pulmonary disorder (COPD), repeating the protocol on each participant six times. The model fitted the data well and parameter values were highly repeatable from test-to-test. Analysis of variance revealed highly significant differences between individuals within groups, and between groups for all three parameters—the individual values for *σ* for *all* COPD patients were *always* higher than those for any of the control participants. As an example, Fig. 3 shows the distributions for standardized compliance, *C*_L_*, and conductance, *C*_d_*, for each participant group, and there is a clear increase in the width of the distributions for the COPD group. Furthermore, the distribution of dead space was found to be markedly larger for COPD patients. These initial results indicate that such measurements are capable of *stratifying* lung disease [44].

#### Optical feedback cavity-enhanced absorption spectroscopy

Optical feedback cavity-enhanced absorption spectroscopy (OF-CEAS) solves one of the most pertinent problems of CEAS implementations, the difficulty of injecting a sufficient amount of laser light into a very high-finesse optical cavity, while providing a good spectral resolution and high signal-to-noise ratio. This spectroscopic method has been described in detail in several publications [45–47]. Here, we just present its basic principles. Like the other CEAS methods, OF-CEAS achieves a high sensitivity by the use of a resonant optical cavity as the sample cell. The effective absorption path length can then easily reach tens of kilometers, with a very compact setup. The originality of OF-CEAS is that the optical cavity is made of three mirrors placed in a “V-shaped” configuration (Fig. 1 in [45]). In this way, a fraction of the light trapped inside the optical cavity, and therefore frequency-selected by the cavity, can be returned to the laser. The nonlinear response of the laser is then exploited to force lasing on the exact frequency of the excited cavity “mode”. This “optical feedback” (OF) effect is also responsible for a narrowing of the laser emission line width with a consequent increase of the cavity transmission together with a reduction of the noise (which normally results from the coupling of narrow cavity modes with a relatively broader laser line). The resulting signal-to-noise ratio is orders of magnitude larger than in other spectroscopic methods [47]. The cavity length is typically 1 m (folded to an external base length of just 50 cm), while the effective absorption path length easily reaches tens of kilometers.

Using DFB diode lasers or ICLs/QCLs, OF-CEAS absorption spectra are acquired in a small spectral region (~ 1.5 GHz), by scanning the laser frequency at a relatively fast rate (~ 10 Hz). However, the response time is not limited by this rate but rather by the gas exchange inside the measurement volume. Therefore, the cell is designed with minimal dead space and a small sample volume of less than 20 cm^3^. The gas sample can be injected in a continuous flow with a stabilized, relatively low cell pressure, and its exchange time depends on the flow and pressure. It can be made below 1 s, for example, by setting a flow of 8 mL/s with a cavity pressure of 140 mbar [46]. If required, it can be further improved using a lower pressure and/or a higher pumping rate. Finally, the design of the spectrometer is robust and compact: the optical assembly and all the electronics for real-time control and data acquisition fit inside a 19″ chassis.

Successful inter-comparison of OF-CEAS analyzers and mass spectrometer [48] as well as gas chromatograph have been reported [49]. A real-time numerical fit of the measured absorption spectra enables the selective determination of the concentrations of all compounds that possess absorption lines in the selected spectral window. This is a key point in breath analysis, as expired air has a highly complex gas mixture.

OF-CEAS analyzers have been developed in the NIR since 2005 with DFB diode lasers. The smallest detectable absorption coefficient is typically on the order of 5 × 10^−10^ cm^−1^Hz^−1/2^ in this spectral region [47]. As an example, an OF-CEAS instrument optimized for monitoring exhaled CO at 2.33 µm (4297.7 cm^−1^) offers a 0.2 ppb detection limit for CO over an acquisition time of 20 s [50]. More recently, a few OF-CEAS prototype setups in the MIR were reported based on QCL [51, 52] and lately, based on ICLs [53–55], presenting an absorption detection limit in the range of 10^− 9^ cm^−1^Hz^−1/2^. This level of sensitivity of OF-CEAS analyzers in the mid-IR results directly from its high signal-to-noise ratio compensating for the lower detectivity of optical detectors at longer wavelengths. Reaching this spectral region is of special interest for clinical applications, since it allows trace detection of NO, a well-studied molecule by clinicians. In particular, an OF-CEAS analyzer developed with a QCL at 5.26 µm (1900.5 cm^−1^) achieves a sensitivity of 80 ppt of NO for a single measurement performed in 140 ms. The stability of the instrument allows averaging for 10 s to reach ~ 10 ppt [56].

**Fig. 4:**
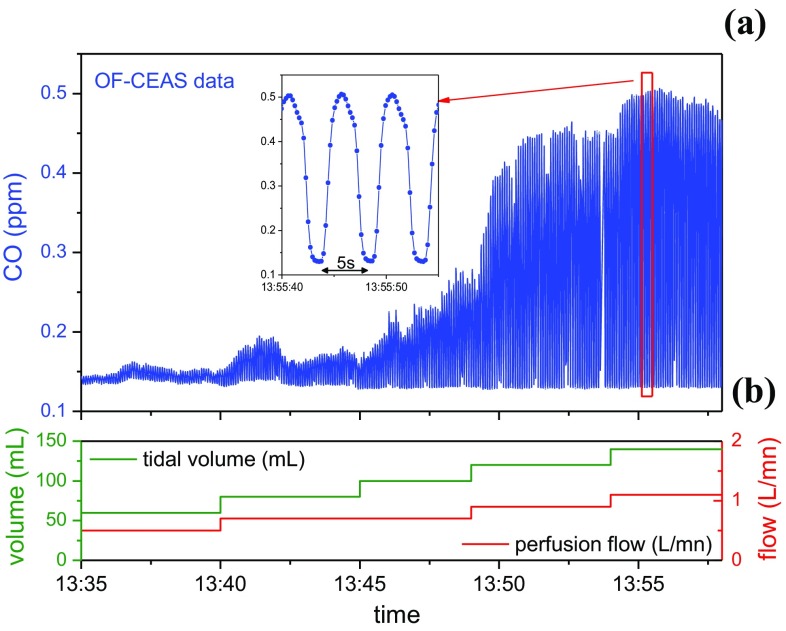
**a** CO measurements performed by OF-CEAS on an ex vivo pig lung during slow warming after cold ischemia. The response time allows to monitor both inspiration and expiration phases imposed by the ventilator (respiratory rate is 12 per min). The baseline corresponds to CO concentration in the medical gas supply. **b** Lungs were progressively ventilated and rewarmed by a perfusion solution

OF-CEAS analyzers in the NIR have been successfully used in two independent medical settings. First, the technique was validated at the Bichat Hospital (Paris, France), monitoring patients’ exhaled CO and methane (CH_4_) in the framework of bronchial inflammation diagnostics. Similar to results presented above in 2.3.1, OF-CEAS measurements were performed simultaneously to spirometer records to trace out levels of CO and CH_4_ during different ventilatory phases [46]. More recently, in collaboration with Grenoble University Hospital, the role of the exhaled CO and NO as biomarkers for the selection of lung grafts for transplantation is being investigated. This study, in the tightly controlled clinical setting of organ transplantation, is driven by the need of new markers for the re-habilitation of pulmonary grafts initially rejected for transplant. Evaluation of isolated lungs occurs in an ex vivo lung perfusion (EVLP) device: lungs are perfused and ventilated in a closed container [57]. As a first demonstration, by analyzing the gas in the ventilator line, the endogenous CO produced by isolated pig lungs was measured by OF-CEAS during the progressive rewarming of the lung after cold ischemia (Fig. 4). These measurements on the isolated organ deliver CO concentrations that are one order of magnitude smaller than measurements performed on the entire living body [58]. In that study, the level of CO production could be correlated to the severity of ischemia–reperfusion injury in EVLP [59], opening the path towards the definition of threshold values of exhaled CO, clinically relevant for EVLP procedures. The monitoring of endogenous gas production for lung graft evaluation is currently on going. First, still on the animal model, NO measurements will be performed. Indeed, NO is a major signaling gas transmitter involved in pulmonary inflammation. It is then very attractive to exploit recent developments of OF-CEAS analyzer in the mid-IR that allow the required sub-ppb sensitivity for NO measurements in isolated lungs [60]. Second, measurements are planned during human lung transplantations.

## Laser photoacoustic spectroscopy

### Principles and overview of photoacoustic spectroscopy

Like other laser spectroscopic methods, laser photoacoustic spectroscopy (LPAS) is able to monitor gas absorptions in a fast and sensitive way. Cavity-enhanced spectroscopic methods derive their high sensitivity from long absorption path lengths (km). Laser photoacoustics has the advantage that the absorption path length is kept very short (mm to cm) and that the signal is background free (i.e., no signal is detected in the absence of absorbers). The method does not rely on a decrease of the transmitted light intensity, but on an increase from a zero baseline, i.e., on release of energy via collisions after absorption. Depopulation of excited energy levels occurs either via fluorescence or collisions. Collisional de-excitation is favorable in the infrared wavelength, due to long fluorescence lifetime in the IR, especially at atmospheric pressures [61]. Due to the transfer of vibrational to translational energy, the temperature of the gas increases. By modulating the radiation source, the temperature will change periodically, giving rise to a periodical pressure change, resulting in an acoustic wave that can be detected by a microphone.

Photoacoustic spectroscopy serves as a very sensitive, efficient, easy, and robust analytical method. In addition, LPAS is relatively cheap and can be used in a very wide wavelength range limited only by the infrared transmittance of the window materials. This is in contrast to CEA, in which the wavelength dependence of the highly reflective mirror coatings limits the spectral coverage of the system. In comparison to direct absorption methods, the photoacoustic signal is proportional to the optical power. From the Lambert–Beer law one finds for small absorptions:

1 $$S~=A \cdot P \cdot \sigma \left( \upsilon \right) \cdot n \cdot l$$

in which *S* is the photoacoustic signal detected by the microphone (in *V*), *P* is the laser power (in *W*), *A* is the conversion efficiency, $\sigma \left( \upsilon \right)$ is the absorption cross section of the molecule (in cm^2^) at frequency $\upsilon$, *n* is the number of molecules per cm^3^, and *l* is the absorption path length (in cm). The constant *A* quantifies the conversion efficiency, which depends on the acoustic geometry of the cell, sensitivity of the microphone, signal amplification, etc.

Due to its high sensitivity, LPAS allows single breath collection from a small sampling volume (few 100 ml) with no pre-concentration steps needed. Within LPAS a high sensitivity can be achieved with high-power infrared lasers. In addition, a wide-tunability is required to selectively detect gases in complex gas mixtures and to operate at the optimal wavelength region to minimize spectroscopic interference from other gases such as water. Such widely tunable high-power lasers are available, for example CO_2_ lasers, optical parametric oscillators (OPOs) [32], or EC-QCLs, the latter being used to determine glucose concentration in human skin [62] and NO detection [63].

An early example illustrating the possibility of LPAS trace gas detection for breath analysis is the investigation of the effect of UV-radiation on the human skin. Under stress conditions (e.g., ionizing radiation, toxic chemical substances, and diseases), the production of free radicals in the body is significantly increased. Subsequently, the capacity of the free radical scavengers in the body is overloaded and a chain of chemical reactions is activated [64]. This leads ultimately to cell membrane damage (i.e., lipid peroxidation, oxidation of fatty acids in cell membrane) that plays an important role in the aging processes and pathogenesis of some diseases. The cell damage is accompanied by the production of light hydrocarbons such as ethane, pentane, and ethylene that can be easily measured with an intracavity CO_2_ laser using photoacoustic lab setup [65, 66].

**Fig. 5:**
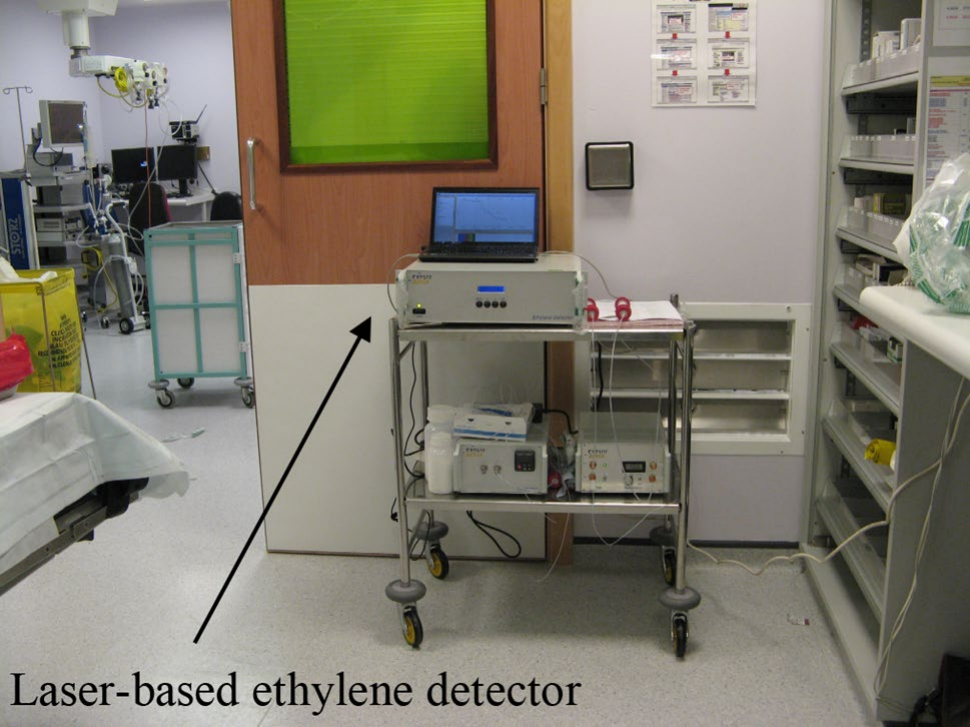
Laser-based photoacoustic detector (ETD-300, Sensor Sense) in the cardiothoracic surgery department at Harefield hospital, UK (photo by S. Cristescu)

**Fig. 6:**
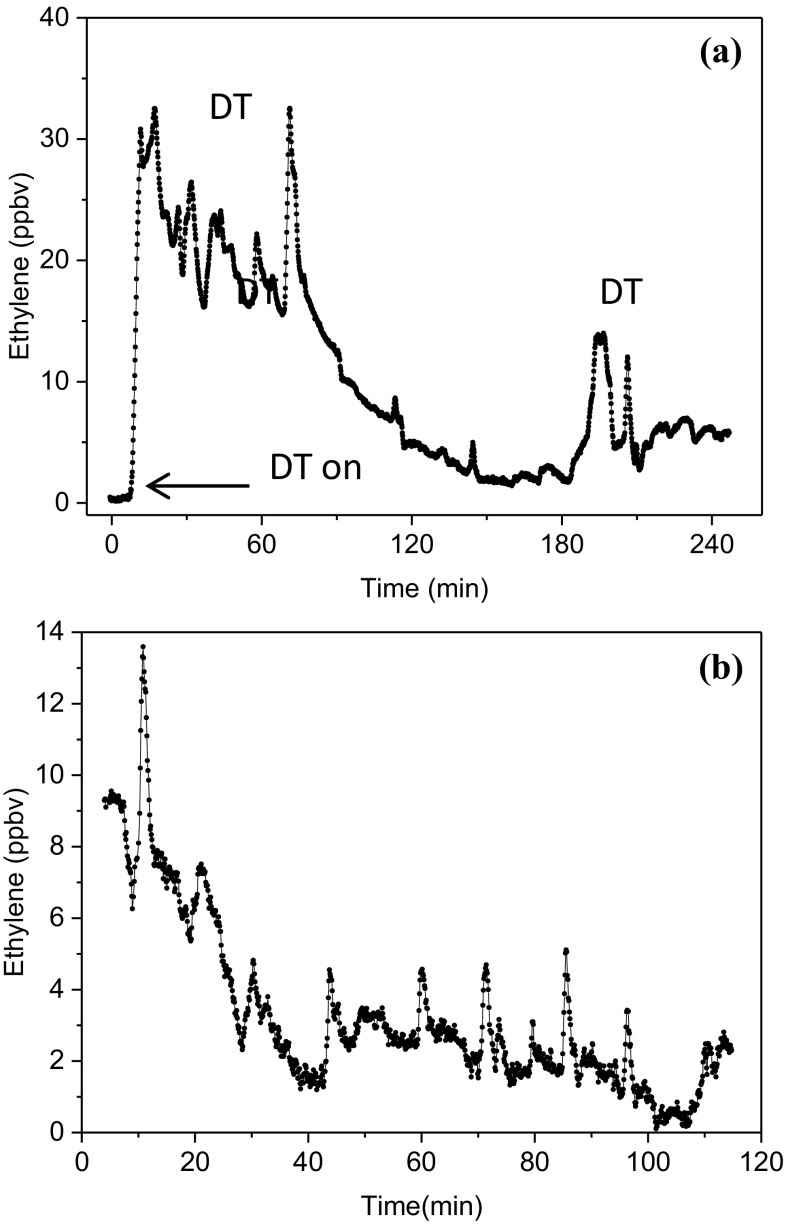
Intraoperative real-time monitoring of ethylene measured by LPAS in patients undergoing off-pump coronary artery bypass surgery. **a** Example of continuous monitoring during of the entire operation. At *t* = 0 h the patient was connected to the sampling line; DT = diathermy electrocautery with high-frequency electric currents (adapted from [67]). **b** An example of a grafting procedure. The high-resolution peaks are associated with ethylene induced by lipid peroxidation during the reperfusion events

This investigation is recently extended to a clinical setting, with the assessment of lipid peroxidation by expired ethylene during cardiac surgery (Fig. 5) using a commercial CO_2_ laser-based photoacoustic detector with a detection limit of 300 ppt in 5 s (Sensor Sense, The Netherlands). During this surgery, patients were undergoing aortic or mitral valve surgery-requiring cardiopulmonary bypass and off-pump coronary artery bypass surgery. The study revealed, for the first time, the real-time signatures of the surgical response triggered by oxidative stress including lipid peroxidation (Fig. 6) [67]. This innovative approach has irrefutably demonstrated the significant contribution of diathermy to overall oxidative stress and identified increased ethylene associated with regional myocardial ischemia on an individual basis. It can be concluded that sensitive, totally non-invasive, and real-time analysis of lipid peroxidation using breath ethylene is feasible in the clinical and perioperative setting [68].

Mid-infrared OPOs are considered amongst the most useful tools for sensitive LPAS gas sensing, due to their high power (Watt level), wide tuning range, and ease of tunability. Such a light source was successfully applied for the detection of hydrogen cyanide (HCN) in exhaled breath of cystic fibrosis (CF) patients being infected with various bacteria, namely *Pseudomonas aeruginosa, Staphylococcus aureus*, etc. [69, 70]. In high concentrations HCN is toxic, but it may also originate endogenously (in small amounts), from pathogens [69–71] or ingestion of food [32]. Infections with these bacteria are of particular significance to CF patients, due to their association with an increased morbidity and mortality. Therefore, it is important to be able to detect these bacterial infections as early as possible. Current diagnostic techniques lack sensitivity (cough swab), are very invasive, and commonly miss early infections, especially in young children.

### Quartz-enhanced photoacoustic spectroscopy

The current field of medical applications demands not only better quantification of exhaled gases with high sensitivity and fast time-response, but also portable devices with low power consumption. Quartz-enhanced photoacoustic spectroscopy (QEPAS) can meet these demands to a large extent. The common approach in gas phase LPAS spectroscopy is to use a resonant photoacoustic cell, but this can be replaced by a tuning fork therefore decreasing the sampling volume. Tuning forks are mass produced and inexpensive; every electronic watch or clock is built around a high-Q quartz crystal frequency standard. The resonance frequency (32 kHz) of the fork corresponds to a symmetric vibration (the prongs move in opposite directions). The antisymmetric vibration is piezoelectrically inactive. A typical tuning fork has at normal atmospheric pressure a Q-value of 8000. Special features are: (a) ambient acoustic noise is low at those frequencies; (b) external sound will not excite the piezoelectrically active mode of the prongs; (c) the width of resonance is 4 Hz (at atmospheric pressure) and only frequency components in this narrow spectral band can produce efficient excitation of the TF vibration; and (d) the gas sampling volume is extremely small (0.15 mm^3^) [72].

There are a number of examples of breath studies using QEPAS, such as: (i) determining the ^13^CO_2_/CO_2_ ratio from human breath for identification of *Helicobacter pylori* infection, liver malfunction, and excessive growth of bacteria in the body [73]; (ii) the detection of carbon disulfide (CS_2_) to indicate cirrhosis and a potential non-invasive marker of respiratory bacterial colonization in cystic fibrosis [74]; and (iii) NH_3_ detection as product of protein metabolism relevant for a number of disease states and human physiology [75].

**Fig. 7:**
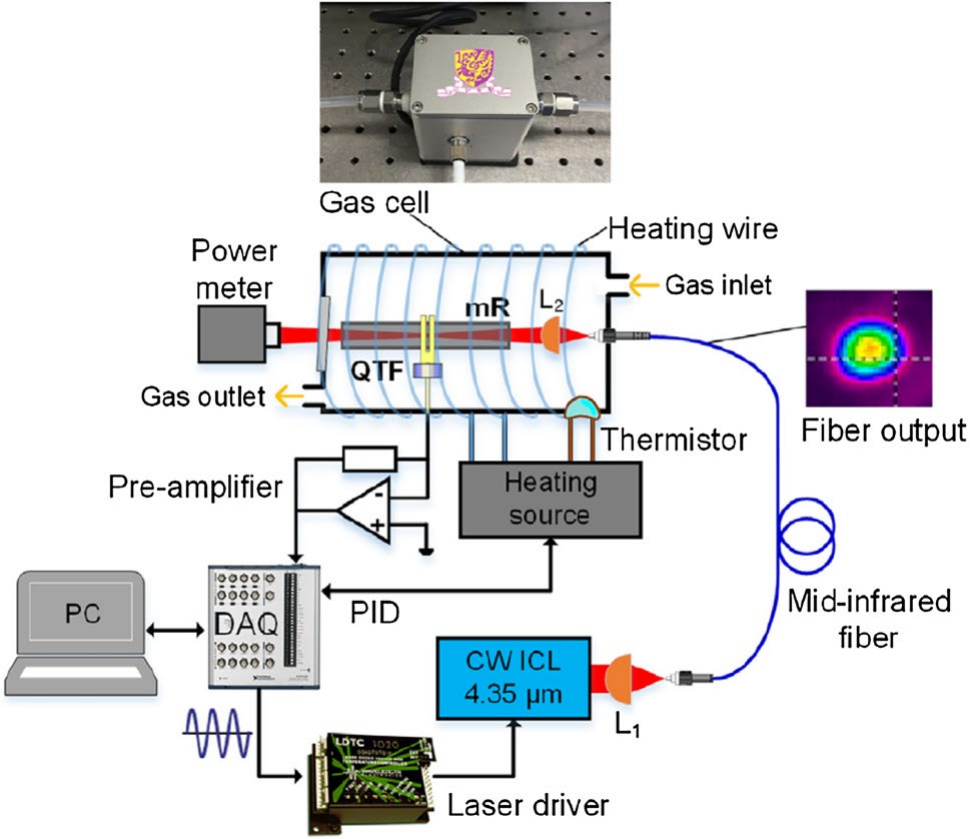
A scheme of the fiber-coupled off-beam QEPAS sensor for CO_2_ isotopic ratio determination [73]. L1, AR-coated aspheric lens; L2, optical focuser; QTF, quartz tuning fork; mR, micro-resonator; DAQ, data acquisition system

To develop a QEPAS system portable, dedicated mid-infrared Interband Cascade Lasers (ICL) or Quantum Cascade Lasers (QCL) can be used. A typical scheme of a fiber-coupled portable QEPAS setup is shown in Fig. 7 [73]. In this setup, light emitted from the ICL is coupled into a solid-core InF_3_ fiber. The fiber output is directed through a stainless-steel micro-resonator tube (a small acoustic resonance cell). A small slit in the middle of the resonator tube couples the resonant acoustic energy into the quartz tuning fork placed next to the resonator tube. All the QEPAS sensing elements were enclosed in a compact gas sensor with a size of 6 × 5 × 6 cm, with miniaturized drivers, amplifiers and software-based signal processing methods. This system was used to determine CO_2_ isotopic ratios with a precision of < 1%.

## Optical frequency comb spectroscopy

An Optical frequency comb (OFC) combines the advantages of narrow linewidth cw lasers with those of broadband supercontinuum light sources. The spectrum of an OFC consists of hundreds of thousands of synchronized coherent modes, spanning a broad wavelength range. This feature makes OFC an ideal source for sensitive, broadband and high-resolution spectroscopy. Their potential in simultaneous detection of different biomarkers in human breath has been already demonstrated in the near-IR wavelength range using cavity-enhanced optical frequency comb spectroscopy (CE-OFCS) [76], where trace concentrations of CO, NH_3_, and methane (CH_4_) in exhaled breath were detected in the presence of high concentrations of water vapor (H_2_O) and carbon dioxide (CO_2_). The distinct combination of broad spectral bandwidth and high spectral resolution of the frequency combs makes it possible to detect molecules with broad absorption features and at the same time discriminate and characterize the interfering species, yielding a selective multispecies detection advantage.

Although most of the important species for biomedical applications have their strongest ro-vibrational transitions in the mid-IR, the OFCs are traditionally generated from the mode-locked lasers whose spectrum usually covers the visible and near-IR wavelength ranges, while reliable mode-lock laser emitting in mid-IR are still under development [77]. To reach the mid-IR wavelength range two main different approaches are most often used [78]: nonlinear frequency conversion such as optical parametric oscillation (OPO) or difference frequency generation (DFG) in combination with near-IR mode-locked lasers, or alternatively generating mid-IR comb directly from semiconductor lasers. Other approaches such as using microresonators to generate mid-IR combs are under development [79].

OFCs based on nonlinear conversion have been used to detect different volatile compounds connected with potential breath analysis applications, such as methane and nitrous oxide (N_2_O) using OPOs with a cavity-enhanced absorption and Fourier Transform Spectrometry (FTS) [80]; CH_4_, formaldehyde (CH_2_O), C_2_H_4_ and CO using intracavity detection with an OPO and FTS [81]; CH_4_ using DFG and an Optical spectrum analyzer (OSA) [82]; and CH_4_, CO and NO using an OPO with a multipass cell and FTS [83]. Different broadband spectroscopic methods such as Virtually Imaged Phase Array (VIPA) spectroscopy [84], Vernier spectroscopy [85], and dual-comb spectroscopy [86, 87] are also utilized in the MIR range yielding faster measurement times and/or higher detection sensitivity paving the way towards achieving the specifications needed for biomedical applications. In addition, the recent dual-comb spectroscopic results using a DFG [88] or an OPO [89] based on an Orientation Patterned Gallium Phosphide (OPGaP) crystal—a newly developed nonlinear material—extend the available detection wavelength range to 12 µm. Despite all of the advantages and recent advances of the mid-IR spectroscopy systems based on nonlinear conversion, they are still complicated, bulky, and expensive; which seems to be the main drawback of utilizing these systems in actual biomedical applications until now.

The second main approach is to use semiconductor mode-locked lasers, specifically QCLs, for generating mid-IR combs [90] and implement a compact dual-comb spectroscopy system [91, 92]. The potential of these spectroscopy systems in detection of different biomarkers such as NH_3_ and N_2_O [91, 93] has been shown; however, their sensitivity needs to be significantly improved, e.g., using enhancement cavities, to make them appropriate for biomedical applications. These systems are commercially available, quite compact, robust and rather inexpensive. However, compared to the mid-IR combs based on nonlinear conversion and mode-locked lasers, the QCL-based combs have typically one to two orders of magnitude narrower spectral bandwidths and higher repetition rates. Narrower spectral bandwidths potentially restrain the multispecies detection capability, and higher repetition rates—assuming no spectral interleaving [94]—limit the spectral resolution of the spectrometers based on these sources making them more appropriate for spectroscopy of liquid and solid samples. A brief comparison between the key features of the available mid-IR spectrometers based on OPO/DFG generated combs and QCL generated combs is presented in Table 1.

**Table 1:** Comparison between the key features of typical mid-IR spectrometers based on OPO/DFG generated combs and QCL generated combs

Specification	mid-IR spectrometers based on OPO/DFG combs	mid-IR spectrometers based on QCL combs
Possible spectral coverage	2.5–12 µm	5–12 µm
Spectral bandwidth	Up to ~ 1500 cm^−1^	Up to ~ 150 cm^−1^
Spectral tunability	Yes	No
Typical repetition rate (spectral sampling)	~ 100 MHz	~ 10 GHz
Average output power	Up to ~ 250 mW	Up to ~ 1 W
Detection sensitivity	High (w/cavity enhancement)	Low (w/o cavity enhancement)
Complexity	High	Low
Size	Bulky	Compact
Cost	Expensive	Inexpensive

## Performance comparison and SWOT analysis

Here, we present a performance comparison of the presented laser-based techniques with typical mass spectrometers and sensor arrays (Table 2), as well as a SWOT analysis (strengths, weaknesses, opportunities, and threats) for the laser-based techniques in the following subsections. The aim is to position the laser spectroscopy within the breath research field, and to give examples of the latest technological advances and applications in clinical environments.

**Table 2:** Performance of LAS techniques as compared to other analytical methods in the field of breath gas analysis, based on included references. Real-time—online sampling and breath-cycle resolved detection, VOCs—volatile organic compounds

Technique	Sensitivity	Selectivity/precision	Real time	Multispecies	Complexity/size/cost	Comments
*Laser based*						
TDLAS and MPC	Intermediate	High	Yes	1–3	Low to intermediate	Light molecules
CEAS	High		Yes	1–3	Intermediate	
PAS	High		No	1–3	Intermediate	
OFCS	Intermediate		Not yet	Yes	High	Light mol. and VOCs
*MS*						
GC-MS	High	Intermediate	No	Yes	High	VOCs, calibration
PTR-MS SIFT-MS	High	Intermediate	Yes	Yes	High	VOCs, calibration
Sensor arrays	Intermediate	Poor	No	–	Low	Calibration

### Strengths

Although the literature on breath analysis seems dominated by mass spectrometry and other non-optical sensors, the breath tests approved by the US Food and Drug Administration [95] include numerous optical-based measurements, such as urea breath test, capnography and F_E_NO (chemiluminescence—“semi-optical”). Interestingly, with one exception, namely the test for heart transplant rejection (C_4_-C_20_ alkanes and branched chain alkanes) [96], most of the FDA approved tests are single-species tests. This is where the strength of optical methods lies in: specific, reliable, and quantitative, real-time analysis of single analytes, and often without the need for (frequent) calibration. In Table 3, representative molecules of medical interest that have been detected in (mostly) breath samples with near- and mid-infrared laser-based spectroscopic methods are listed. Depending on the specific experimental setup, measurements have been performed in real-time (sub-second time resolution, resolving individual breath cycles) or near-real-time (not resolving breath cycles, but analysis complete in time scale of minutes).

**Table 3:** Representative molecules of medical interest determined with laser-based spectroscopic methods in near- and mid-infrared regions

Molecule	*λ* (µm)	Detection method	Detection limit (measurement time)	Human tests/in vitro/application is breath, otherwise specified
Acetaldehyde	5.79	TDLAS/WMS	80 ppb (5 s)	After alcoholic beverage [97]
Acetone	~ 8.2	ICOS	170 ppb (0.2 s)	[98]
	~ 3.3	ICOS	100 ppb (0.4 s)	[99]
	~ 3.3	TDLAS + multipass cell	14 ppb (15 min)	Diabetes [100]
	1.69	CEAS	~ 160 ppb	[101, 102]
	~ 8.2	TDLAS + multipass cell	150 ppb	Diabetes [103]
	~ 8.2	ICOS	30 ppb (30 s)	[104]
Acetylene	1.52	CRDS	175 ppt (10 min)	After smoking [105]
	1.53	ICOS	1.5 ppb (128 s)	After smoking [106]
Ammonia	~ 3	CRDS	0.3 ppb (30 s)	Breath and skin [107, 108], during hemodialysis [109]
	9.56	PAS	100 ppb (3 s)	Hemodialysis [110]
	9.56	QEPAS	10 ppb (1 s)	[111], Exercise [112]
	3.04	PAS	1 ppm (10 s)	Bacteria [70]
	10.3	CRDS	50 ppb (20 s)	[113]
	10.3	TDLAS + multipass cell	3 ppb (10 s)	[114]
	9.07	WMS + multipass cell	7 ppb (5 s)	[115]
	1.51	CE-OFCS	4 ppm (30 s)	[76]
Carbon dioxide (isotopes)	1.60	CRDS	3 ppm (3 ms)	[116]
	1.60	CE-OFCS	< 10 ppm (30 s)	[76]
	2.05	ICOS	± 0.15‰ precision for δ^13^_DOB_^13^C‰	*H. pylori* infection [117, 118]
	~ 2	ICAS	~ 100 ppb	[119]
Carbon monoxide	4.57	TDLAS + multipass cell	0.8 ppb (1 s)	Asthma, COPD [34]
	4.59	TDLAS + multipass cell	14 ppb (60 s)	[120]
	4.72	WMS + multipass cell	0.6 ppb (15 s)	Smoking [36]
	4.61	ICOS/WMS	7 ppb (1 s)	[38]
	2.33	OF-CEAS	1 ppb (0.3 s)	[46]
	1.6	CE-OFCS	0.9 ppm (0.3 s)	[76]
Carbon monoxide (isotopes)	4.69	WMS + multipass cell	0.6 ppb (10 s)	[39]
Carbonyl sulfide	4.86	TDLAS + multipass cell	1.2 ppb (0.4 s)	[121]
	4.90	CRDS	7 ppt (1 s)	[122]
Ethane	3.35	ICOS	0.48 ppb (1 s)	[123]
	3.34	ICOS	50 ppt (0.25 s)	[99]
	3.34	TDLAS/WMS + multipass cell	70 ppt (1 s)	Dialysis [40, 124, 125]
	~ 3.4	CRDS	0.5 ppb (0.8 s)	[126]
	~ 3.4	TDLAS + multipass cell	70 ppt (0.7 s)	[127], Lung cancer [128]
	3.34	CRDS	270 ppt (1 s)	[129]
Ethylene	10.5	PAS	0.3 ppb (5 s)	Cardiac surgery [67], Endotoxemia [130], skin [66]
	10.5	PAS	100 ppb	Renal failure [131]
Glucose	~ 9	PAS	~ 30 mg/dL (in blood)	Skin [62]
Hydrogen cyanide	1.54	CRDS	0.3 ppb (2.5 min)	Breath, skin [132], Oral fluid [133]
	3.04	PAS	0.4 ppb (10 s)	[69], in vitro [70]
Methane	1.65	PAS	0.3 ppm (12 s)	[134]
	2.33	OF-CEAS	25 ppb (0.3 s)	[46]
	~ 3.3	CRDS	1–10 ppb (120 s)	[135]
Nitric oxide	5.23	ICOS + WMS	2 ppb (15 s)	[136]
	5.22	TDLAS + multipass cell	2 ppb (1 s)	[137]
	5.22	TDLAS + multipass cell	1.5 ppb (4 s)	[138]
	5.26	TDLAS + multipass cell	0.5 ppb (1 s)	Asthma, COPD [34], Septic shock [139]
	5.22	ICOS	0.4 ppb (1 s)	[140]
	5.2	ICOS	0.7 ppb (1 s)	Breath and skin [141]
Nitric oxide (isotopes)	5.33	CRDS	7 ppt (70 s)	[142]
	5.43	Faraday rotation spectroscopy	0.5–4 ppb (1 s)	Breath, blood, urine [143]
Nitrous oxide	4.57	TDLAS + multipass cell	0.8 ppb (1 s)	Asthma, COPD [34]

Recent advances in nanotechnologies, optoelectronics, and photonics integration reinforce the demand for compactness and miniaturization of the devices. Non-specific sensors assembled into arrays that respond to different odors by generating a complex signal, e.g., the electronic nose or semiconductor-based sensor array, have become popular and have been used in clinical applications [144–146]. They are relatively inexpensive, small, easy-to-use, but lack both sensitivity and selectivity, require frequent calibrations, drift over time, have memory effects, are sensitive to changes in humidity and temperature and cannot identify individual compounds [25, 147]. Moreover, since a limited number of specific sensorial elements are used, it is mandatory to know which compounds will be targeted and/or the composition matrix of the background [144–146]. Optical techniques are by far more sensitive and selective, are virtually maintenance free and can operate continuously for long periods of time. For example, in comparison with a traditional electrochemical CO sensor (used to access smoking status), TDLAS-based CO detection (see Sect. 3.1) offers much better sensitivity, precision and time resolution, and thus is better suited to study the CO physiology.

Importantly, OF-CEAS provides quantitative absorption measurements in real time without the need for periodic calibration (using certified gas mixtures). A normalization procedure of the absorbance scale is realized by performing a single CRDS measurement at the end of each OF-CEAS spectral scan [148]. In addition, OF-CEAS is an ultra-sensitive technique allowing selective real-time measurements using a small sampling volume with the response time faster than a single respiratory cycle. Since it enables the development of compact instruments to be operated by non-specialists in a medical environment it also offer commercially attractive commercial perspective (e.g., OF-CEAS analyzers are currently commercialized by AP2E, France).

### Weaknesses

In contrast to standard analytical techniques (e.g., GC-MS), laser-based spectroscopy is mainly used in research focused on a single identifiable molecule or a small combination of molecules whose biochemistries are known. Simultaneous multispecies detection in breath with optical methods has only been demonstrated for few species at a time, at trace levels (ppb and below).

Indeed, in the exploratory/discovery phase of breath research, instrumentation that can probe only single analyte is not (necessarily) sufficient. In many cases it would be useful to have an instrument that can analyze many volatile compounds. And this is where mass spectrometry still holds an advantage over optical methods. The gold standard in breath research is gas chromatography combined with mass spectrometry (GC-MS) [149]. GC-MS allows the unambiguous identification of hundreds of volatile breath compounds and the method has been used extensively in exhaled breath studies. However, it is not suitable for online sampling and the analysis is not real time. Some form of pre-concentration (adsorbent traps, solid-phase micro-extraction) is required and standard analysis time is up to tens of minutes. These limitations can be circumvented using soft chemical ionization instead of electron impact ionization. Instruments based on selected ion flow-tube mass spectrometry (SIFT-MS) and proton-transfer reaction mass spectrometry (PTR-MS) allow online sampling and real-time analysis while making it potentially possible to analyses dozens of VOCs simultaneously [150].

Few studies have carefully addressed the untargeted analysis of medium-to-high molecular weight species that absorb over a broad wavelength range [151]. As outlined in Sect. 5, demonstration experiments have been conducted using optical frequency combs, but practical routine implementations lie still in the future. However, once the technology matures, optical methods can start competing with real-time MS-based techniques such as PTR-MS, SIFT-MS, and SESI-MS, which are able to measure a broad range of VOCs. When broadband emission sources are used, such as EC-QCLs or OFCs, the main challenge is to develop robust algorithms that can discriminate between multiple absorbing molecules using their specific (narrowband or broadband) spectroscopic features. Even so, the existing possibility of scanning very fast over a broad spectral range to obtain a molecular fingerprint of an unprecedented large number of gases with optical techniques is well worth exploring.

### Opportunities

The technology of lasers has advanced considerably in the past few years and more wavelengths are available for gas sensing and applications outside the laboratory. Furthermore, new avenues have been opened for the design of compact instruments. This has been demonstrated by examples presented in this article. The latest development of QEPAS (Sect. 4.2) allows the design of a gas sensor of less than 220 cm^3^. The MFS approach described in Sect. 3.2.1 is practical in a clinical setting and has many attractive features in comparison to other methods for studying lung inhomogeneity: it is simple to undertake, it is non-invasive, it does not require ionizing radiation (unlike CT and PET), it does not require expensive scanners and reagents (unlike He and Xe-MRI), and it is sufficiently simple to conduct that it could be undertaken in any standard lung-function testing laboratory.

Simultaneous in situ multicomponent measurements next to high sensitivity and fast response time (ms to µs) are important requirements for breath analysis. Few spectroscopic techniques are able to fulfill these demands and a new promising candidate is the intracavity absorption spectroscopy (ICAS). Using a broadband tunable Tm/Ho-doped fiber laser (4780–5560 cm^−1^) and placing the sample inside the cavity, simultaneous detection of H_2_O, ^12^CO_2_, ^13^CO_2_ and ^16^O^12^C^18^O in breath was shown [119].

Furthermore, also broadband absorbing molecules, such as acetone [152], can be detected in breath samples with a portable and compact system. For example, Hancock et al. have demonstrated lab-based mid- and near-IR CEAS systems for breath acetone detection [98, 101]. Most recently, the same group have reported on a portable and compact device for measuring acetone in breath samples; the device features a 7 cm long high-finesse optical cavity that is coupled to a miniature adsorption pre-concentrator containing 0.5 g of polymer material [102]. Acetone is trapped out of breath and released into the optical cavity where it is probed by a near-IR diode laser operating at ~ 1670 nm. With an optical cavity mirror reflectivity of 99.994%, a precision of 100 ppb is achieved with direct breath sampling. The method was validated with measurements made using a chemical ionization mass spectrometer and measurements on individuals under fasting, exercise, and normal conditions have been presented, indicating the utility of the device across a wide dynamic range. The device has a sufficiently small power requirement that battery operation is possible for a reasonable number of measurements. This device has ready applicability for using breath acetone analysis to provide an alternative to blood testing for ketone measurement, to assist with the management of type 1 diabetes [153].

The fast development of the QCL combs, e.g., achieving higher power levels [154] and improvement in their frequency stability [155, 156] promises substantial improvements in the operation of QCL-based dual-comb spectrometers in the near future, making them more interesting for biomedical applications.

It is well known that the time to market a device for medical purposes is longer than for other fields of applications. This might explain why some optical methods are not yet on the market, even though they have shown large potentials, as demonstrated by the examples presented here.

For detection of some compounds, such as C_2_H_4_ or NH_3_, optical methods are preferred over the MS-based ones, due to a better sensitivity, simplicity in operation and instrument price. Also, the ability of soft-ionization MS to detect light molecules is limited to those species with sufficient proton affinity. Notably, NH_3_ cannot be sampled off-line, due to its “sticky” nature and interaction with surfaces of materials [107, 157].

Exhaled NO (or fractional exhaled NO in the gas phase, F_E_NO) is a well-known biomarker for airway inflammation [158], playing an important role in asthma phenotyping and management [159]. Moreover, its concentration is dependent on the exhalation flow (higher at low flow and vice versa). Studies showed that measuring NO at one flow rate, typically 50 ml/s, can neither determine the alveolar site/peripheral contribution, nor quantify the difference in NO diffusion from the airways walls. The use of NO modeling approach (linear or nonlinear) can solve this issue and provide useful information about the source of NO [2, 160]. There are still many unexplored areas for F_E_NO analysis and examining these will require the implementation of a sampling system allowing different flow rates into the measuring instrument. Different schemes using QCL have been already developed for F_E_NO and some are used in the clinical environment [34]. This will provide a great value in diagnostic procedures of respiratory diseases and in treatment with anti-inflammatory drugs by predicting the inhaled corticosteroid (ICS) response.

The sampling of exhaled breath is as important as its analysis [161, 162]. A careful selection of the laser source for spectroscopic measurements is usually required to limit spectral overlap with water and CO_2_ which are quite abundant in breath. However, whenever possible, it would actually be beneficial to monitor CO_2_ simultaneously with the molecule of interest within the same scan [132, 133, 141]. A CO_2_-controlled sampling of expiratory air allows a proper determination of the end-tidal metabolite concentration.

### Threats

When broad band emission sources are used, such as EC-QCL or OFC, the main challenge is to develop a robust algorithm that can discriminate between multiple absorbing molecules using their specific spectroscopic features. Here, optical techniques will need to compete with other real-time MS-based techniques such as PTR-MS, SIFT-MS, and SESI-MS.

The latest incremental developments in MS-based technology seem to strengthen their market position. A representative example is PTR-MS; although the instruments are not becoming cheaper, on the contrary, their features look potentially attractive for multispecies real-time monitoring. Over time, the PTR-MS instruments have been equipped with a time-of-flight (TOF) analyzer to improve the mass resolution and enable the analysis of compounds with almost the same molecular mass. The PTR-MS instruments have also been incorporated with additional reagent ions (O_2_+, NO+, Kr + or Xe + in addition to standard H_3_O^+^) which allow the ionization and detection of a larger variety of volatile compounds. Recently, a hexapole ion guide and a short chromatographic column (FastGC) are also available with the PTR-MS instruments. The main benefit of coupling a FastGC to a PTR-MS is that it allows the detection of isomers due to the chromatographic separation of the compounds prior to entering the PTR-MS [163]. Furthermore, commercial PTR-MS instruments now include software that allows convenient data analysis of real-time measurements of breath profiles. This is of great importance since it aids in the identification of the different respiratory phases (dead space, end-tidal) by measuring the time-profiles of specific breath volatiles (e.g., acetone). The need for an elaborate breath sampler (incorporating a CO_2_ analyzer, for example) can thus be circumvented by multispecies real-time analysis.

Other techniques are finding their way into breath analysis; several clinical trials are presently ongoing with GC-IMS and SESI-MS. The question is whether this could change the market beyond the ability of the optical techniques to adapt and secure their position.

For detection of exhaled compounds in high concentration (ppm and above), such as methane, the electrochemical sensors are highly competitive with the laser-based techniques, especially that they are already commercially available from various vendors [164]. Furthermore, an ingestible electronic capsule based on semiconducting metal oxide-based sensor has demonstrated its capability of sensing O_2_, H_2_ and CO_2_ in the gut during a human pilot trial [165]. Using CRD technique with a Q-switched Nd:YAG laser at 266 nm, a standalone portable breath acetone analyzer was developed and tested on diabetic patients [166].

## Current progress and challenges

With the technological developments been stimulated by the growing evidence of clinical worth, advanced research moved from measuring relevant molecules in laboratory to pilot studies in hospitals. The transition to clinical practice is in continuous progress; more commercial instruments found their way into clinics and reveal unprecedented information, with the few examples presented here. Like any other techniques, to get into routine practice, the optical techniques need to overcome still several barriers at various levels from instrumental and scientific hurdles to regulatory approvals and financials [23, 27].

*Breath sampling and measurement*. At this stage, a lot of the practical problems regarding breath sampling and measurement have been identified and solutions are in place. This is still a crucial aspect for further implementation, not only for the optical techniques, but also for MS-based ones. Researchers became more aware of the importance of controlling the exhalation rate and monitoring the exhaled CO_2_ in any accessible manner; most measurements are designed for acquisition of the alveolar air and elimination of the dead air space. No matter whether the measurements are online or offline, these set of parameters are the first steps towards standardization of breath sampling. Lessons have been learned by experience; therefore, rather than still being named a challenge, standardization includes a series of protocols that have been proposed and are currently tested for validation. For example, off-line collection (usually in Tedlar bags) requires a proper choice for the material and additional tests to check the cleanliness of the bags and the stability over time of the analyte of interest. For single or multiple exhalations, reproducibility of the breath samples needs to be also checked.

*Instrumental challenge*. Compact and user-friendly instruments are highly desired, providing accurate measurements, preferably single time-point breath collection with high sensitivity and specificity. They should monitor changes in biomarker concentrations or parameters compared to control or baseline values, etc. Therefore, calibration of the measuring system for determination of the gas concentration needs to be done taking into account the main compounds present in the matrix of breath, namely H_2_O and CO_2_ in air.

*Scientific challenge and outcomes*. Most of the compounds that can be measured with the laser-based techniques have known origin and biochemical pathways. Furthermore, understanding the connection biomarker-disease-health conditions, and response of biomarker to interventions (exposure, isotope labeling, therapy, etc.) is crucial for integration into clinical practice. The high precision and absolute accuracy offered by the laser-based techniques allows collection of more data and in consequence, very detailed biomarker studies. Small differences can be thus easier resolved, gas exchange in the respiratory tract can be characterized so that the origin and chemical pathways can be better understood. In turn, it will become more clear which biomarker reliably correlates with diseases. Another aspect to be considered is that variability of the results may be strongly influenced by diet and life style and exposure to various environmental conditions.

*Marketing and financial aspects*. The success of a new breath test on the medical market depends on how well it is marketed to patients, physicians, heath institutions, and insurance companies.

## Concluding remarks and future perspectives

Numerous laser spectroscopy methods have been developed in the laboratory during several decades and are reaching maturity. Their potential for medical analysis, in particular expired air, have been recognized early and numerous laboratory demonstrations have been published. Today we are at a turning point, where techniques which have become widely used tools for the laser spectroscopists, can be promoted in new domains, in particular for medical applications. For that, it is necessary that researchers specialized in laser spectroscopy meet medical specialists to engage together in the instrumental development needed for their specific implementation (or application) in medical research. The rapidly increasing interest from the medical community, thanks to the recent promising findings in a wide range of (clinical) applications, is supporting breath analysis to become even more clinically available in the future, hopefully at least partly replacing current blood-based bioassays.

## References

[1] R. Lewicki, A.A. Kosterev, D.M. Thomazy, T.H. Risby, S. Solga, T.B. Schwartz, F.K. Tittel, Real time ammonia detection in exhaled human breath using a distributed feedback quantum cascade laser based sensor. in *SPIE OPTO*(SPIE2011), p. 7

[2] Cristescu SM, Mandon J, Harren FJM, Merilainen P, Hogman M (2013). Methods of NO detection in exhaled breath. J. Breath Res..

[3] Amann A, Costello B, Miekisch W, Schubert J, Buszewski B, Pleil J, Ratcliffe N, Risby T (2014). The human volatilome: volatile organic compounds (VOCs) in exhaled breath, skin emanations, urine, feces and saliva. J. Breath Res..

[4] Costello BD, Amann A, Al-Kateb H, Flynn C, Filipiak W, Khalid T, Osborne D, Ratcliffe NM (2014). A review of the volatiles from the healthy human body. J. Breath Res..

[5] Pleil D, Stiegel MA, Risby TH (2013). Clinical breath analysis: discriminating between human endogenous compounds and exogenous (environmental) chemical confounders. J. Breath Res..

[6] Stönner C, Williams J (2016). Goals change crowd air chemistry. Nature.

[7] Turner MA, Bandelow S, Edwards L, Patel P, Martin HJ, Wilson ID, Thomas CLP (2013). The effect of a paced auditory serial addition test (PASAT) intervention on the profile of volatile organic compounds in human breath: a pilot study. J. Breath Res..

[8] M.K. Paschke Kelly, Clinical applications of breath testing. F1000 Medicine Reports 2 (2010) 10.3410/M2-56 PMC2990505 21173863

[9] Pauling L, Robinson AB, Teranish R, Cary P (1971). Quantitative analysis of urine vapor and breath by gas-liquid partition chromatography. Proc. Natl. Acad. Sci. U. S. A..

[10] A. Amann, W. Miekisch, J. Schubert, B. Buszewski, T. Ligor, T. Jezierski, J. Pleil, T. Risby, Analysis of exhaled breath for disease detection. in *Annual Review of Analytical Chemistry, Vol 7*, ed. by R.G. Cooks, J.E. Pemberton, (Annual Reviews, 2014), pp. 455–482 10.1146/annurev-anchem-071213-020043 25014347

[11] Basanta M, Ibrahim B, Dockry R, Douce D, Morris M, Singh D, Woodcock A, Fowler SJ (2012). Exhaled volatile organic compounds for phenotyping chronic obstructive pulmonary disease: a cross-sectional study. Respir. Res..

[12] Miekisch W, Herbig J, Schubert JK (2012). Data interpretation in breath biomarker research: pitfalls and directions. J. Breath Res..

[13] Phillips M, Cataneo RN, Chaturvedi A, Kaplan PD, Libardoni M, Mundada M, Patel U, Zhang X (2013). Detection of an extended human volatome with comprehensive two-dimensional gas chromatography time-of-flight mass spectrometry. Plos ONE.

[14] Schnabel R, Fijten R, Smolinska A, Dallinga J, Boumans ML, Stobberingh E, Boots A, Roekaerts P, Bergmans D, van Schooten FJ (2015). Analysis of volatile organic compounds in exhaled breath to diagnose ventilator-associated pneumonia. Sci. Rep..

[15] del Rio RF, O’Hara ME, Holt A, Pemberton P, Shah T, Whitehouse T, Mayhew CA (2015). Volatile biomarkers in breath associated with liver cirrhosis—comparisons of pre-and post-liver transplant breath samples. Ebiomedicine.

[16] Dryahina K, Smith D, Bortlik M, Machkova N, Lukas M, Spanel P (2018). Pentane and other volatile organic compounds, including carboxylic acids, in the exhaled breath of patients with Crohn’s disease and ulcerative colitis. J. Breath Res..

[17] Mochalski P, Wiesenhofer H, Allers M, Zimmermann S, Güntner AT, Pineau NJ, Lederer W, Agapiou A, Mayhew CA, Ruzsanyi V (2018). Monitoring of selected skin- and breath-borne volatile organic compounds emitted from the human body using gas chromatography ion mobility spectrometry (GC-IMS). J. Chromatogr. B.

[18] Gaugg MT, Gomez DG, Barrios-Collado C, Vidal-de-Miguel G, Kohler M, Zenobi R, Sinues PML (2016). Expanding metabolite coverage of real-time breath analysis by coupling a universal secondary electrospray ionization source and high resolution mass spectrometry-a pilot study on tobacco smokers. J. Breath Res..

[19] Kibion, Kibion Dynamic. http://kibion.com/product-range/kibion-dynamic/

[20] Bedfont, GastroCH_4_ECK. https://www.bedfont.com/shop/gastrolyzer/GastroCH4ECK

[21] LaserBreath, LaserBreath001. https://laserbreathtechnology.com/products/laserbreath001

[22] Lourenço C, Turner C (2014). Breath analysis in disease diagnosis: methodological considerations and applications. Metabolites.

[23] Risby TH, Tittel FK (2010). Current status of midinfrared quantum and interband cascade lasers for clinical breath analysis. Opt. Eng..

[24] Stacewicz T, Bielecki Z, Wojtas J, Magryta P, Mikolajczyk J, Szabra D (2016). Detection of disease markers in human breath with laser absorption spectroscopy. Opto-Electron. Rev..

[25] Wang CJ, Sahay P (2009). Breath analysis using laser spectroscopic techniques: breath biomarkers, spectral fingerprints, and detection limits. Sensors.

[26] Wojtas J, Bielecki Z, Stacewicz T, Mikolajczyk J, Nowakowski M (2012). Ultrasensitive laser spectroscopy for breath analysis. Opto-Electron. Rev..

[27] Modak AS (2011). Barriers to overcome for transition of breath tests from research to routine clinical practice. J. Breath Res..

[28] Alving K, Weitzberg E, Lundberg JM (1993). Increased amount of nitric oxide in exhaled air of asthmatics. Eur. Respir. J..

[29] Maniscalco M, Vitale C, Vatrella A, Molino A, Bianco A, Mazzarella G (2016). Fractional exhaled nitric oxide-measuring devices: technology update. Med. Devices (Auckland, N.Z.).

[30] Gisbert JP, Pajares JM (2004). ^13^C-urea breath test in the diagnosis of Helicobacter pylori infection—a critical review. Aliment. Pharmacol. Ther..

[31] Modak AS (2018). Point-of-care companion diagnostic tests for personalizing psychiatric medications: fulfilling an unmet clinical need. J. Breath Res..

[32] Arslanov DD, Castro MPP, Creemers NA, Neerincx AH, Spunei M, Mandon J, Cristescu SM, Merkus P, Harren FJM (2013). Optical parametric oscillator-based photoacoustic detection of hydrogen cyanide for biomedical applications. J. Biomed. Opt..

[33] Hannemann M, Antufjew A, Borgmann K, Hempel F, Ittermann T, Welzel S, Weltmann KD, Volzke H, Ropcke J (2011). Influence of age and sex in exhaled breath samples investigated by means of infrared laser absorption spectroscopy. J. Breath Res..

[34] Shorter JH, Nelson DD, McManus JB, Zahniser MS, Sama SR, Milton DK (2011). Clinical study of multiple breath biomarkers of asthma and COPD (NO, CO_2_, CO and N_2_O) by infrared laser spectroscopy. J. Breath Res..

[35] Ciaffoni L, O’Neill DP, Couper JH, Ritchie GA, Hancock G, Robbins PA (2016). In-airway molecular flow sensing: a new technology for continuous, noninvasive monitoring of oxygen consumption in critical care. Sci. Adv..

[36] Ghorbani R, Schmidt FM (2017). Real-time breath gas analysis of CO and CO_2_ using an EC-QCL. Appl. Phys. B-Lasers Opt..

[37] Bartlome R, Sigrist MW (2009). Laser-based human breath analysis: D/H isotope ratio increase following heavy water intake. Opt. Lett..

[38] Pakmanesh N, Cristescu SM, Ghorbanzadeh A, Harren FJM, Mandon J (2016). Quantum cascade laser-based sensors for the detection of exhaled carbon monoxide. Appl. Phys. B-Lasers Opt..

[39] Ghorbani R, Schmidt FM (2017). ICL-based TDLAS sensor for real-time breath gas analysis of carbon monoxide isotopes. Opt. Express.

[40] Patterson CS, McMillan LC, Longbottom C, Gibson GM, Padgett MJ, Skeldon KD (2007). Portable optical spectroscopy for accurate analysis of ethane in exhaled breath. Meas. Sci. Technol..

[41] Ciaffoni L, Couper J, Hancock G, Peverall R, Robbins PA, Ritchie GAD (2014). RF noise induced laser perturbation for improving the performance of non-resonant cavity enhanced absorption spectroscopy. Opt. Express.

[42] Manfred KM, Kirkbride JMR, Ciaffoni L, Peverall R, Ritchie GAD (2014). Enhancing the sensitivity of mid-IR quantum cascade laser-based cavity-enhanced absorption spectroscopy using RF current perturbation. Opt. Lett..

[43] O’Neill DP, Robbins PA (2017). A mechanistic physicochemical model of carbon dioxide transport in blood. J. Appl. Physiol..

[44] J.E. Mountain, P. Santer, D.P. O’Neill, N.M.J. Smith, L. Ciaffoni, J.H. Couper, G.A.D. Ritchie, G. Hancock, J.P. Whiteley, P.A. Robbins, Potential for non-invasive assessment of lung inhomogeneity using highly precise, highly time-resolved, measurements of gas exchange J. Appl. Physiol. 0, jap.00745.02017 (2017) 10.1152/japplphysiol.00745.2017 PMC5899273 29074714

[45] Morville J, Kassi S, Chenevier M, Romanini D (2005). Fast, low-noise, mode-by-mode, cavity-enhanced absorption spectroscopy by diode-laser self-locking. Appl. Phys. B-Lasers Opt..

[46] Ventrillard-Courtillot I, Gonthiez T, Clerici C, Romanini D (2009). Multispecies breath analysis faster than a single respiratory cycle by optical-feedback cavity-enhanced absorption spectroscopy. J. Biomed. Opt..

[47] Morville J, Romanini D, Kerstel E, Gagliardi G, Loock H-P (2014). Cavity enhanced absorption spectroscopy with optical feedback. Cavity-Enhanced Spectroscopy and Sensing.

[48] R.Q. Iannone, D. Romanini, O. Cattani, H.A.J. Meijer, E.R.T. Kerstel, Water isotope ratio (δ^2^H and δ^18^O) measurements in atmospheric moisture using an optical feedback cavity enhanced absorption laser spectrometer. J. Geophys. Res. Atmos. **115**, 1AA (2010)

[49] Ventrillard I, Xueref-Remy I, Schmidt M, Kwok CY, Fain X, Romanini D (2017). Comparison of optical-feedback cavity-enhanced absorption spectroscopy and gas chromatography for ground-based and airborne measurements of atmospheric CO concentration. Atmos. Meas. Tech..

[50] Fain X, Chappellaz J, Rhodes RH, Stowasser C, Blunier T, McConnell JR, Brook EJ, Preunkert S, Legrand M, Debois T, Romanini D (2014). High resolution measurements of carbon monoxide along a late Holocene Greenland ice core: evidence for in situ production. Clim. Past.

[51] Hamilton DJ, Orr-Ewing AJ (2011). A quantum cascade laser-based optical feedback cavity-enhanced absorption spectrometer for the simultaneous measurement of CH4 and N2O in air. Appl. Phys. B-Lasers Opt..

[52] Maisons G, Carbajo PG, Carras M, Romanini D (2010). Optical-feedback cavity-enhanced absorption spectroscopy with a quantum cascade laser. Opt. Lett..

[53] Manfred KM, Hunter KM, Ciaffoni L, Ritchie GAD (2017). ICL-based OF-CEAS: a sensitive tool for analytical chemistry. Anal. Chem..

[54] Manfred KM, Ritchie GAD, Lang N, Röpcke J, Van Helden JH (2015). Optical feedback cavity-enhanced absorption spectroscopy with a 3.24 µm interband cascade laser. Appl. Phys. Lett..

[55] Richard L, Ventrillard I, Chau G, Jaulin K, Kerstel E, Romanini D (2016). Optical-feedback cavity-enhanced absorption spectroscopy with an interband cascade laser: application to SO_2_ trace analysis. Appl. Phys. B-Lasers Opt..

[56] Ventrillard I, Gorrotxategi-Carbajo P, Romanini D (2017). Part per trillion nitric oxide measurement by optical feedback cavity-enhanced absorption spectroscopy in the mid-infrared. Appl. Phys. B-Lasers Opt..

[57] Cypel M, Yeung JC, Liu MY, Anraku M, Chen FS, Karolak W, Sato M, Laratta J, Azad S, Madonik M, Chow CW, Chaparro C, Hutcheon M, Singer LG, Slutsky AS, Yasufuku K, de Perrot M, Pierre AF, Waddell TK, Keshavjee S (2011). Normothermic ex vivo lung perfusion in clinical lung transplantation. N. Engl. J. Med..

[58] Maignan M, Briot R, Romanini D, Gennai S, Hazane-Puch F, Brouta A, Debaty G, Ventrillard I (2014). Real-time measurements of endogenous carbon monoxide production in isolated pig lungs. J. Biomed. Opt..

[59] Maignan M, Gennai S, Debaty G, Romanini D, Schmidt MH, Brenckmann V, Brouta A, Ventrillard I, Briot R (2017). Exhaled carbon monoxide is correlated with ischemia reperfusion injuries during ex vivo lung perfusion in pigs. J. Breath Res..

[60] Marczin N, Riedel B, Gal J, Polak J, Yacoub M (1997). Exhaled nitric oxide during lung transplantation.. Lancet.

[61] Harren FJM, Mandon J, Cristescu SM, Meyers RA (2012). Photoacoustic spectroscopy in trace gas monitoring. Encyclopedia of Analytical Chemistry.

[62] Pleitez MA, Lieblein T, Bauer A, Hertzberg O, von Lilienfeld-Toal H, Mantele W (2013). In vivo noninvasive monitoring of glucose concentration in human epidermis by mid-infrared pulsed photoacoustic spectroscopy. Anal. Chem..

[63] Spagnolo V, Kosterev AA, Dong L, Lewicki R, Tittel FK (2010). NO trace gas sensor based on quartz-enhanced photoacoustic spectroscopy and external cavity quantum cascade laser. Appl. Phys. B-Lasers Opt..

[64] Kneepkens CMF, Lepage G, Roy CC (1994). The potential of the hydrocarbon breath test as a measure of lipid peroxidation. Free Radic. Biol. Med..

[65] Harren F, Berkelmans R, Kuiper K, te Lintel Hekkert S, Scheepers P, Dekhuijzen R, Hollander P, Parker D (1999). On-line laser photoacoustic detection of ethene in exhaled air as biomarker of ultraviolet radiation damage of the human skin. Appl. Phys. Lett..

[66] Moeskops BWM, Steeghs MML, van Swam K, Cristescu SM, Scheepers PTJ, Harren FJM (2006). Real-time trace gas sensing of ethylene, propanal and acetaldehyde from human skin in vivo. Physiol. Meas..

[67] Cristescu SM, Kiss R, Hekkert ST, Dalby M, Harren FJM, Risby TH, Marczin N, Harefield BSI (2014). Real-time monitoring of endogenous lipid peroxidation by exhaled ethylene in patients undergoing cardiac surgery. Am. J. Physiol.-Lung Cell. Mol. Physiol..

[68] R. Romano, S.M. Cristescu, T.H. Risby, N. Marczin, Lipid peroxidation in cardiac surgery: towards consensus on biomonitoring, diagnostic tools and therapeutic implementation J. Breath Res. 12 (2018) 10.1088/1752-7163/aa9856 29104182

[69] Neerincx AH, Linders YAM, Vermeulen L, Belderbos RA, Mandon J, van Mastrigt E, Pijnenburg MW, van Ingen J, Mouton JW, Kluijtmans LAJ, Wevers RA, Harren FJM, Cristescu SM, Merkus P (2016). Hydrogen cyanide emission in the lung by Staphylococcus aureus. Eur. Respir. J..

[70] Neerincx AH, Mandon J, van Ingen J, Arslanov DD, Mouton JW, Harren FJ, Merkus PJ, Cristescu SM (2015). Real-time monitoring of hydrogen cyanide (HCN) and ammonia (NH_3_) emitted by Pseudomonas aeruginosa. J. Breath Res..

[71] Chen W, Roslund K, Fogarty CL, Pussinen PJ, Halonen L, Groop PH, Metsala M, Lehto M (2016). Detection of hydrogen cyanide from oral anaerobes by cavity ring down spectroscopy. Sci. Rep..

[72] Kosterev AA, Tittel FK, Serebryakov DV, Malinovsky AL, Morozov IV (2005). Applications of quartz tuning forks in spectroscopic gas sensing. Rev. Sci. Instrum..

[73] Wang Z, Wang Q, Ching JYL, Wu JCY, Zhang GF, Ren W (2017). A portable low-power QEPAS-based CO_2_ isotope sensor using a fiber-coupled interband cascade laser. Sens. Actuators B Chem..

[74] Waclawek JP, Moser H, Lendl B (2016). Compact quantum cascade laser based quartz-enhanced photoacoustic spectroscopy sensor system for detection of carbon disulfide. Opt. Express.

[75] Spacek LA, Mudalel M, Tittel F, Risby TH, Solga SF (2015). Clinical utility of breath ammonia for evaluation of ammonia physiology in healthy and cirrhotic adults. J. Breath Res..

[76] Thorpe MJ, Balslev-Clausen D, Kirchner MS, Ye J (2008). Cavity-enhanced optical frequency comb spectroscopy: application to human breath analysis. Opt. Express.

[77] Duval S, Gauthier JC, Robichaud LR, Paradis P, Olivier M, Fortin V, Bernier M, Piche M, Vallee R (2016). Watt-level fiber-based femtosecond laser source tunable from 2.8 to 3.6 µm. Opt. Lett..

[78] Schliesser A, Picque N, Hansch TW (2012). Mid-infrared frequency combs. Nat. Photonics.

[79] Yu MJ, Okawachi Y, Griffith AG, Lipson M, Gaeta AL (2017). Microresonator-based high-resolution gas spectroscopy. Opt. Lett..

[80] Foltynowicz A, Maslowski P, Fleisher AJ, Bjork BJ, Ye J (2013). Cavity-enhanced optical frequency comb spectroscopy in the mid-infrared application to trace detection of hydrogen peroxide. Appl. Phys. B-Lasers Opt..

[81] Haakestad MW, Lamour TP, Leindecker N, Marandi A, Vodopyanov KL (2013). Intracavity trace molecular detection with a broadband mid-IR frequency comb source. J. Opt. Soc. Am. B Opt. Phys..

[82] Cruz FC, Maser DL, Johnson T, Ycas G, Klose A, Giorgetta FR, Coddington I, Diddams SA (2015). Mid-infrared optical frequency combs based on difference frequency generation for molecular spectroscopy. Opt. Express.

[83] Khodabakhsh A, Ramaiah-Badarla V, Rutkowski L, Johansson AC, Lee KF, Jiang J, Mohr C, Fermann ME, Foltynowicz A (2016). Fourier transform and Vernier spectroscopy using an optical frequency comb at 3–5.4 µm. Opt. Lett..

[84] Nugent-Glandorf L, Neely T, Adler F, Fleisher AJ, Cossel KC, Bjork B, Dinneen T, Ye J, Diddams SA (2012). Mid-infrared virtually imaged phased array spectrometer for rapid and broadband trace gas detection. Opt. Lett..

[85] Khodabakhsh A, Rutkowski L, Morville J, Foltynowicz A (2017). Mid-infrared continuous-filtering Vernier spectroscopy using a doubly resonant optical parametric oscillator. Appl. Phys. B-Lasers Opt..

[86] Zhang ZW, Gardiner T, Reid DT (2013). Mid-infrared dual-comb spectroscopy with an optical parametric oscillator. Opt. Lett..

[87] Jin YW, Cristescu SM, Harren FJM, Mandon J (2014). Two-crystal mid-infrared optical parametric oscillator for absorption and dispersion dual-comb spectroscopy. Opt. Lett..

[88] H. Timmers, A. Kowligy, A. Lind, F.C. Cruz, N. Nader, M. Silfies, T.K. Allison, G. Ycas, P.G. Schunemann, S.B. Papp, S.A. Diddams, Dual frequency comb spectroscopy in the molecular fingerprint region. arXiv:1712.09764 [physics.optics] (2017)

[89] Kara O, Maidment L, Gardiner T, Schunemann PG, Reid DT (2017). Dual-comb spectroscopy in the spectral fingerprint region using OPGaP optical parametric oscillators. Opt. Express.

[90] Hugi A, Villares G, Blaser S, Liu HC, Faist J (2012). Mid-infrared frequency comb based on a quantum cascade laser. Nature.

[91] Wang Y, Soskind MG, Wang W, Wysocki G (2014). High-resolution multi-heterodyne spectroscopy based on Fabry–Perot quantum cascade lasers. Appl. Phys. Lett..

[92] Villares G, Hugi A, Blaser S, Faist J (2014). Dual-comb spectroscopy based on quantum-cascade-laser frequency combs. Nat. Commun..

[93] Westberg J, Sterczewski LA, Wysocki G (2017). Mid-infrared multiheterodyne spectroscopy with phase-locked quantum cascade lasers. Appl. Phys. Lett..

[94] Baumann E, Giorgetta FR, Swann WC, Zolot AM, Coddington I, Newbury NR (2011). Spectroscopy of the methane u_3_ band with an accurate midinfrared coherent dual-comb spectrometer. Phys. Rev. A.

[95] FDA, FDAMedical Devices. https://www.fda.gov/MedicalDevices/default.htm

[96] Phillips M, Boehmer JP, Cataneo RN, Cheema T, Eisen HJ, Fallon JT, Fisher PE, Gass A, Greenberg J, Kobashigawa J, Mancini D, Rayburn B, Zucker MJ (2004). Heart allograft rejection: detection with breath alkanes in low levels (the HARDBALL study). J. Heart Lung Transpl..

[97] Kamat PC, Roller CB, Namjou K, Jeffers JD, Faramarzalian A, Salas R, McCann PJ (2007). Measurement of acetaldehyde in exhaled breath using a laser absorption spectrometer. Appl. Opt..

[98] Ciaffoni L, Hancock G, Harrison JJ, van Helden J-PH, Langley CE, Peverall R, Ritchie GAD, Wood S (2013). Demonstration of a mid-infrared cavity enhanced absorption spectrometer for breath acetone detection. Anal. Chem..

[99] Arslanov DD, Swinkels K, Cristescu SM, Harren FJM (2011). Real-time, subsecond, multicomponent breath analysis by optical parametric oscillator based off-axis integrated cavity output spectroscopy. Opt. Express.

[100] Tuzson B, Looser H, Felder F, Bovey F, Tappy L, Emmenegger L (2018). Human breath acetone analysis by mid-IR laser spectroscopy: development and application. High-brightness sources and light-driven interactions.

[101] Hancock G, Langley CE, Peverall R, Ritchie GAD, Taylor D (2014). Laser-based method and sample handling protocol for measuring breath acetone. Anal. Chem..

[102] Blaikie TPJ, Couper J, Hancock G, Hurst PL, Peverall R, Richmond G, Ritchie GAD, Taylor D, Valentine K (2016). Portable device for measuring breath acetone based on sample preconcentration and cavity enhanced spectroscopy. Anal. Chem..

[103] Reyes-Reyes A, Horsten RC, Urbach HP, Bhattacharya N (2015). Study of the exhaled acetone in type 1 diabetes using quantum cascade laser spectroscopy. Anal. Chem..

[104] Centeno R, Mandon J, Harren FJM, Cristescu SM (2016). Influence of ethanol on breath acetone measurements using an external cavity quantum cascade laser. Photonics.

[105] Metsala M, Schmidt FM, Skytta M, Vaittinen O, Halonen L (2010). Acetylene in breath: background levels and real-time elimination kinetics after smoking. J. Breath Res..

[106] Marchenko D, Neerincx AH, Mandon J, Zhang J, Boerkamp M, Mink J, Cristescu SM, Hekkert ST, Harren FJM (2015). A compact laser-based spectrometer for detection of C_2_H_2_ in exhaled breath and HCN in vitro. Appl. Phys. B-Lasers Opt..

[107] Schmidt FM, Vaittinen O, Metsala M, Lehto M, Forsblom C, Groop PH, Halonen L (2013). Ammonia in breath and emitted from skin. J. Breath Res..

[108] Chen W, Metsala M, Vaittinen O, Halonen L (2014). The origin of mouth-exhaled ammonia. J. Breath Res..

[109] Chen W, Laiho S, Vaittinen O, Halonen L, Ortiz F, Forsblom C, Groop PH, Lehto M, Metsala M (2016). Biochemical pathways of breath ammonia (NH_3_) generation in patients with end-stage renal disease undergoing hemodialysis. J. Breath Res..

[110] Narasimhan LR, Goodman W, Patel CKN (2001). Correlation of breath ammonia with blood urea nitrogen and creatinine during hemodialysis. Proc. Natl. Acad. Sci. U. S. A..

[111] Lewicki R, Kosterev AA, Bakhirkin YA, Thomazy DM, Doty J, Dong L, Tittel FK, Risby TH, Solga S, Kane D, Day T (2009). Real time ammonia detection in exhaled human breath with a quantum cascade laser based sensor. *Conference on Lasers and Electro*-*Optics*/*International Quantum Electronics Conference*.

[112] Solga SF, Mudalel M, Spacek LA, Lewicki R, Tittel FK, Loccioni C, Russo A, Ragnoni A, Risby TH (2014). Changes in the concentration of breath ammonia in response to exercise: a preliminary investigation. J. Breath Res..

[113] Manne J, Sukhorukov O, Jager W, Tulip J (2006). Pulsed quantum cascade laser-based cavity ring-down spectroscopy for ammonia detection in breath. Appl. Opt..

[114] Manne J, Jager W, Tulip J (2009). Sensitive detection of ammonia and ethylene with a pulsed quantum cascade laser using intra and interpulse spectroscopic techniques. Appl. Phys. B-Lasers Opt..

[115] Owen K, Farooq A (2014). A calibration-free ammonia breath sensor using a quantum cascade laser with WMS 2f/1f. Appl. Phys. B-Lasers Opt..

[116] Crosson ER, Ricci KN, Richman BA, Chilese FC, Owano TG, Provencal RA, Todd MW, Glasser J, Kachanov AA, Paldus BA, Spence TG, Zare RN (2002). Stable isotope ratios using cavity ring-down spectroscopy: determination of ^13^C/^12^C for carbon dioxide in human breath. Anal. Chem..

[117] Maity A, Som S, Ghosh C, Banik GD, Daschakraborty SB, Ghosh S, Chaudhuri S, Pradhan M (2014). Oxygen-18 stable isotope of exhaled breath CO_2_ as a non-invasive marker of Helicobacter pylori infection. J. Anal. At. Spectrom..

[118] Som S, De A, Banik GD, Maity A, Ghosh C, Pal M, Daschakraborty SB, Chaudhuri S, Jana S, Pradhan M (2015). Mechanisms linking metabolism of Helicobacter pylori to ^18^O and ^13^C-isotopes of human breath CO_2_. Sci. Rep..

[119] Fjodorow P, Hellmig O, Baev VM (2018). A broadband Tm/Ho-doped fiber laser tunable from 1.8 to 2.09 mu m for intracavity absorption spectroscopy. Appl. Phys. B-Lasers Opt..

[120] Moeskops BWM, Naus H, Cristescu SM, Harren FJM (2006). Quantum cascade laser-based carbon monoxide detection on a second time scale from human breath. Appl. Phys. B-Lasers Opt..

[121] Wysocki G, McCurdy M, So S, Weidmann D, Roller C, Curl RF, Tittel FK (2004). Pulsed quantum-cascade laser-based sensor for trace-gas detection of carbonyl sulfide. Appl. Opt..

[122] Halmer D, von Basum G, Hering P, Murtz M (2005). Mid-infrared cavity leak-out spectroscopy for ultrasensitive detection of carbonyl sulfide. Opt. Lett..

[123] Parameswaran KR, Rosen DI, Allen MG, Ganz AM, Risby TH (2009). Off-axis integrated cavity output spectroscopy with a mid-infrared interband cascade laser for real-time breath ethane measurements. Appl. Opt..

[124] Patterson CS, McMillan LC, Stevenson K, Radhakrishnan K, Shiels PG, Padgett MJ, Skeldon KD (2007). Dynamic study of oxidative stress in renal dialysis patients based on breath ethane measured by optical spectroscopy. J. Breath Res..

[125] Skeldon KD, Patterson C, Wyse CA, Gibson GM, Padgett MJ, Longbottom C, McMillan LC (2005). The potential offered by real-time, high-sensitivity monitoring of ethane in breath and some pilot studies using optical spectroscopy. J. Opt. A-Pure Appl. Opt..

[126] von Basum G, Dahnke H, Halmer D, Hering P, Murtz M (2003). Online recording of ethane traces in human breath via infrared laser spectroscopy. J. Appl. Physiol..

[127] Skeldon KD, Gibson GM, Wyse CA, McMillan LC, Monk SD, Longbottom C, Padgett MJ (2005). Development of high-resolution real-time sub-ppb ethane spectroscopy and some pilot studies in life science. Appl. Opt..

[128] Skeldon KD, McMillan LC, Wyse CA, Monk SD, Gibson G, Patterson C, France T, Longbottom C, Padgett MJ (2006). Application of laser spectroscopy for measurement of exhaled ethane in patients with lung cancer. Respir. Med..

[129] Halmer D, Thelen S, Hering P, Murtz M (2006). Online monitoring of ethane traces in exhaled breath with a difference frequency generation spectrometer. Appl. Phys. B-Lasers Opt..

[130] Paardekooper LM, van den Bogaart G, Kox M, Dingjan I, Neerincx AH, Bendix MB, Beest Mt, Harren FJM, Risby T, Pickkers P, Marczin N, Cristescu SM (2017). Ethylene, an early marker of systemic inflammation in humans. Sci. Rep..

[131] Popa C, Patachia M, Banita S, Matei C, Bratu AM, Dumitras DC (2013). The level of ethylene biomarker in the renal failure of elderly patients analyzed by photoacoustic spectroscopy. Laser Phys..

[132] Schmidt FM, Metsala M, Vaittinen O, Halonen L (2011). Background levels and diurnal variations of hydrogen cyanide in breath and emitted from skin. J. Breath Res..

[133] Chen W, Metsala M, Vaittinen O, Halonen L (2014). Hydrogen cyanide in the headspace of oral fluid and in mouth-exhaled breath. J. Breath Res..

[134] Tuboly E, Szabo A, Eros G, Mohacsi A, Szabo G, Tengolics R, Rakhely G, Boros M (2013). Determination of endogenous methane formation by photoacoustic spectroscopy. J. Breath Res..

[135] Keppler F, Schiller A, Ehehalt R, Greule M, Hartmann J, Polag D (2016). Stable isotope and high precision concentration measurements confirm that all humans produce and exhale methane. J. Breath Res..

[136] Bakhirkin YA, Kosterev AA, Roller C, Curl RF, Tittel FK (2004). Mid-infrared quantum cascade laser based off-axis integrated cavity output spectroscopy for biogenic nitric oxide detection. Appl. Opt..

[137] Namjou K, Roller CB, Reich TE, Jeffers JD, McMillen GL, McCann PJ, Camp MA (2006). Determination of exhaled nitric oxide distributions in a diverse sample population using tunable diode laser absorption spectroscopy. Appl. Phys. B-Lasers Opt..

[138] Roller C, Namjou K, Jeffers JD, Camp M, Mock A, McCann PJ, Grego J (2002). Nitric oxide breath testing by tunable-diode laser absorption spectroscopy: application in monitoring respiratory inflammation. Appl. Opt..

[139] Oever J, Mandon J, Netea MG, van Deuren M, Harren FJM, Cristescu SM, Pickkers P (2013). Pulmonary infection, and not systemic inflammation, accounts for increased concentrations of exhaled nitric oxide in patients with septic shock. J. Breath Res..

[140] McCurdy MR, Bakhirkin Y, Wysocki G, Tittel FK (2007). Performance of an exhaled nitric oxide and carbon dioxide sensor using quantum cascade laser-based integrated cavity output spectroscopy. J. Biomed. Opt..

[141] Marchenko D, Mandon J, Cristescu SM, Merkus PJFM, Harren FJM (2013). Quantum cascade laser-based sensor for detection of exhaled and biogenic nitric oxide. Appl. Phys. B.

[142] Heinrich K, Fritsch T, Hering P, Murtz M (2009). Infrared laser-spectroscopic analysis of ^14^NO and ^15^NO in human breath. Appl. Phys. B-Lasers Opt..

[143] Wang Y, Nikodem M, Zhang E, Cikach F, Barnes J, Comhair S, Dweik RA, Kao C, Wysocki G (2015). Shot-noise limited Faraday rotation spectroscopy for detection of nitric oxide isotopes in breath, urine, and blood. Sci Rep.

[144] Di Natale C, Paolesse R, Martinelli E, Capuano R (2014). Solid-state gas sensors for breath analysis: a review. Anal. Chim. Acta.

[145] Leopold JH, Bos LDJ, Sterk PJ, Schultz MJ, Fens N, Horvath I, Bikov A, Montuschi P, Di Natale C, Yates DH, Abu-Hanna A (2015). Comparison of classification methods in breath analysis by electronic nose. J. Breath Res..

[146] Rock F, Barsan N, Weimar U (2008). Electronic nose: current status and future trends. Chem. Rev..

[147] Righettoni M, Tricoli A, Gass S, Schmid A, Amann A, Pratsinis SE (2012). Breath acetone monitoring by portable Si:WO_3_ gas sensors. Anal. Chim. Acta.

[148] Kerstel ERT, Iannone RQ, Chenevier M, Kassi S, Jost HJ, Romanini D (2006). A water isotope (^2^H, ^17^O, and ^18^O) spectrometer based on optical feedback cavity-enhanced absorption for in situ airborne applications. Appl. Phys. B.

[149] Amann A, Ligor M, Ligor T, Bajtarevic A, Ager C, Pienz M, Denz H, Fiegl M, Hilbe W, Weiss W, Lukas P, Jamnig H, Hackl M, Haidenberger A, Sponring A, Filipiak W, Miekisch W, Schubert J, Troppmair J, Buszewski B (2010). Analysis of exhaled breath for screening of lung cancer patients. Memo. Mag. Eur. Med. Oncol..

[150] Smith D, Spanel P, Herbig J, Beauchamp J (2014). Mass spectrometry for real-time quantitative breath analysis. J. Breath Res..

[151] Mastrigt E, Reyes-Reyes A, Brand K, Bhattacharya N, Urbach HP, Stubbs AP, de Jongste JC, Pijnenburg MW (2016). Exhaled breath profiling using broadband quantum cascade laser-based spectroscopy in healthy children and children with asthma and cystic fibrosis. J. Breath Res..

[152] Ruzsanyi V, Kalapos MP (2017). Breath acetone as a potential marker in clinical practice. J. Breath Res..

[153] Blaikie TPJ, Edge JA, Hancock G, Lunn D, Megson C, Peverall R, Richmond G, Ritchie GAD, Taylor D (2014). Comparison of breath gases, including acetone, with blood glucose and blood ketones in children and adolescents with type 1 diabetes. J. Breath Res..

[154] Jouy P, Wolf JM, Bidaux Y, Allmendinger P, Mangold M, Beck M, Faist J (2017). Dual comb operation of λ~8.2 µm quantum cascade laser frequency comb with 1 W optical power. Appl. Phys. Lett..

[155] Cappelli F, Campo G, Galli I, Consolino L, Giusfredi G, Cancio P, Borri S, Hinkov B, Mazzotti D, Bartalini S, Faist J, Natale PD (2017). Towards the full frequency stabilization of quantum cascade laser frequency combs. *2017 European Conference on Lasers and Electro*-*Optics and European Quantum Electronics Conference*.

[156] Cappelli F, Campo G, Galli I, Giusfredi G, Bartalini S, Mazzotti D, Cancio P, Borri S, Hinkov B, Faist J, De Natale P (2016). Frequency stability characterization of a quantum cascade laser frequency comb. Laser Photonics Rev..

[157] Vaittinen O, Metsälä M, Persijn S, Vainio M, Halonen L (2014). Adsorption of ammonia on treated stainless steel and polymer surfaces. Appl. Phys. B.

[158] Piers RB, George BH, Nandor M (2013). Exhaled nitric oxide as biomarker of acute lung injury: an unfulfilled promise?. J. Breath Res..

[159] Essat M, Harnan S, Gomersall T, Tappenden P, Wong R, Pavord I, Lawson R, Everard ML (2016). Fractional exhaled nitric oxide for the management of asthma in adults: a systematic review. Eur. Respir. J..

[160] Hogman M (2012). Extended NO analysis in health and disease. J. Breath Res..

[161] Lawal O, Ahmed WM, Nijsen TME, Goodacre R, Fowler SJ (2017). Exhaled breath analysis: a review of ‘breath-taking’ methods for off-line analysis. Metabolomics.

[162] Miekisch W, Kischkel S, Sawacki A, Liebau T, Mieth M, Schubert JK (2008). Impact of sampling procedures on the results of breath analysis. J. Breath Res..

[163] Romano A, Fischer L, Herbig J, Campbell-Sills H, Coulon J, Lucas P, Cappellin L, Biasioli F (2104). Wine analysis by FastGC proton-transfer reaction-time-of-flight-mass spectrometry. Int. J. Mass Spectrom..

[164] Costello B, Ledochowski M, Ratcliffe NM (2013). The importance of methane breath testing: a review. J. Breath Res..

[165] Kalantar-Zadeh K, Berean KJ, Ha N, Chrimes AF, Xu K, Grando D, Ou JZ, Pillai N, Campbell JL, Brkljača R, Taylor KM, Burgell RE, Yao CK, Ward SA, McSweeney CS, Muir JG, Gibson PR (2018). A human pilot trial of ingestible electronic capsules capable of sensing different gases in the gut. Nat. Electron..

[166] Sun MX, Jiang CY, Gong ZY, Zhao XM, Chen ZY, Wang ZN, Kang ML, Li YX, Wang CJ (2015). A fully integrated standalone portable cavity ringdown breath acetone analyzer. Rev. Sci. Instrum..

